# Eye Movement Traces of Linguistic Knowledge in Native and Non-Native Reading

**DOI:** 10.1162/opmi_a_00084

**Published:** 2023-06-05

**Authors:** Yevgeni Berzak, Roger Levy

**Affiliations:** Faculty of Data and Decision Sciences, Technion - Israel Institute of Technology, Haifa, Israel; Department of Brain and Cognitive Sciences, Massachussets Institute of Technology, Cambridge, MA

**Keywords:** eye movements, reading, language learning, L1, L2

## Abstract

The detailed study of eye movements in reading has shed considerable light into how language processing unfolds in real time. Yet eye movements in reading remain inadequately studied in non-native (L2) readers, even though much of the world’s population is multilingual. Here we present a detailed analysis of the quantitative functional influences of word length, frequency, and predictability on eye movement measures in reading in a large, linguistically diverse sample of non-native English readers. We find many similar qualitative effects as in L1 readers, but crucially also a proficiency-sensitive “*lexicon-context tradeoff*”. The most proficient L2 readers’ eye movements approach an L1 pattern, but as L2 proficiency diminishes, readers’ eye movements become less sensitive to a word’s predictability in context and more sensitive to word frequency, which is context-invariant. This tradeoff supports a rational, experience-dependent account of how context-driven expectations are deployed in L2 language processing.

## INTRODUCTION

Eye movements in reading provide fine-grained information about how language understanding unfolds in real time in the human mind, and present one of the most detailed pictures of the perception–inference–action cycle in human behavior for complex informational domains (Just & Carpenter, 1980; Rayner, 1998). The large majority of work in eye movements in reading focuses on native-language (L1) reading. However, many of the people in the world are multilingual, and a large amount of reading is done in non-native (L2) languages, making L2 reading an under-studied area. This is especially the case for English, where about 75% of English speakers are not native (Crystal, 2003).

One of the most significant advances in eye movements research over the past several decades has been the development of quantitative models of the relationship between linguistic properties of words and eye movements in reading (Kliegl et al., 2004; Rayner et al., 2004, 2011, among others). A key finding of this line of work is the identification of three key linguistic properties of words, often referred to as “benchmark” word properties or the “big three”, which systematically explain substantial variance in mean fixation times: word length, word frequency and word predictability. These effects have been shown to apply across languages, and their functional form has been studied in L1 (Kliegl et al., 2004; Smith & Levy, 2013; Wilcox et al., 2020). However, only a few studies have examined benchmark word property effects in L2 reading (Cop, Keuleers, Drieghe, & Duyck, 2015; Mor & Prior, 2022; Whitford & Titone, 2012, 2017), and both their functional form in L2 and their relation to language proficiency are currently unknown.

Here, we address these gaps by conducting a quantitative investigation of benchmark word property effects in English L2 reading and compare them to English L1 reading. Our analysis is performed at a scale and level of detail greater than previously possible, due to the introduction of CELER (Berzak et al., 2022), a large and linguistically diverse sample of L2 reading. CELER has 69 L1 participants and 296 L2 participants from five typologically diverse native language backgrounds: Arabic, Chinese, Japanese, Portuguese and Spanish. Differently from other eye movements in L2 reading datasets such as GECO (Cop, Drieghe, & Duyck, 2015) and MECO-L2 (Kuperman et al., 2022), CELER includes scores on a standardized English proficiency test. This facilitates a comprehensive characterization of the trajectory of benchmark word property effects in L2 reading as a function of English proficiency.

More broadly, our study poses the following key question: how does the role of linguistic context in generating expectations during reading vary depending on a comprehender’s language proficiency? We address this question within a theoretical framework of rational processing efficiency. This framework predicts that an optimal system might use contextualized expectations less than context-independent expectations the lower the language proficiency of the speaker. One reason for this is that context-contingent expectations are statistically intrinsically harder to estimate than context-independent expectations. Therefore, the less language experience the speaker has, the more they might rely on more reliable context-independent expectations. Another reason is that context-contingent expectations likely are computationally more difficult to deploy—they have to be updated in real time as the context evolves, and the speed of expectation deployment may be lower when the speaker has less experience with a language. Accordingly, less proficient speakers may need to rely more on context-independent expectations that are easier to estimate and deploy.

We test this prediction by taking advantage of the fact that word frequency and word predictability effects manifest ubiquitously and strongly during reading. Based on previous work (Howes & Solomon, 1951; Smith & Levy, 2013), we operationalize these measures as negative log-frequency and negative log-predictability, or *surprisal*. We compare frequency and surprisal effects in L2 versus L1 readers of English, and in readers of varying L2 proficiency, using standard fixation measures of progressively longer duration, thereby supporting a detailed comparison between participants as online language processing unfolds over time. We perform three analyses in which we examine the *functional form* of benchmark word property effects, their *magnitude*, and *how they depend on language proficiency*. These analyses build on prior work that estimates frequency and predictability effects in L1 and L2 using linear modeling (Cop, Keuleers, et al., 2015; Mor & Prior, 2022; Whitford & Titone, 2012, 2017). We go beyond this prior work by characterizing the functional form of these effects, explicitly comparing them to one another, estimating their dependence on language proficiency using standardized test scores, and using a larger and more linguistically diverse dataset.

## METHODS

### Dataset

We use the CELER dataset (Berzak et al., 2022) which contains 365 participants (296 L2 and 69 L1). The L2 participants come from five different L1s: Arabic, Chinese, Japanese, Portuguese and Spanish. Each participant in CELER reads 156 randomly selected sentences from the Wall Street Journal (WSJ) (Charniak et al., 2000; Marcus et al., 1993). Of these, 78 sentences are unique to each participant (Individual Regime), and 78 are presented to all participants (Shared Regime).

To encourage attentive reading, upon completion of reading each sentence participants answered a yes/no question about its content, and were subsequently informed if they answered the question correctly. The 78 questions for the Shared Regime sentences are reading comprehension questions that were composed manually by the experimenters. The questions for the Individual Regime sentences were generated automatically, and ask whether a given word appeared in the sentence. Figure S10 in the Supplemental Materials depicts the scores of the L2 participants on all the 156 questions against their MPT English proficiency scores described below. All but one participant have above chance performance on these questions.

All the L2 participants were assessed for English proficiency in lab using the listening comprehension and grammar sections of the Michigan English Placement Test (MPT) Form B. The test materials have 50 multiple choice questions, with 20 listening comprehension questions and 30 written grammar questions. The test score is computed as the number of correct answers for these questions, with possible scores ranging from 0 to 50. The test scores correspond to CEFR levels (Council of Europe, 2001) approximately as follows: 0–16 A1, 17–21 A2, 22–31 B1, 32–36 B2, 37–50 C1 (Berzak et al., 2022).

To avoid overfitting to a small set of sentences, we use the Individual Regime materials, comprising 28,457 sentences and 320,221 words. Following standard practice, we exclude out-of-vocabulary words, skipped words, words with punctuation, numbers, and words that begin or end a sentence. This leads to a total of 28,099 sentences and 181,448 words used in our analyses.

### Word Property Annotations

Each word *w*_*i*_ in CELER is annotated with its negative log-frequency (negative log-unigram probability): −log_2_ *p*(*w*_*i*_), surprisal: −log_2_ *p*(*w*_*i*_|*w*_1_, …, *w*_*i*−1_) and word length. Frequency counts are taken from the standard frequency list SUBTLEX-US (Brysbaert & New, 2009). Surprisal values are computed using the state-of-the-art language model GPT2 (Radford et al., 2019). In cases where the GPT tokenizer splits a word into multiple tokens, we sum the surprisal values of those tokens. Word length values exclude punctuation.

### GAM Model

**Figure 1.**
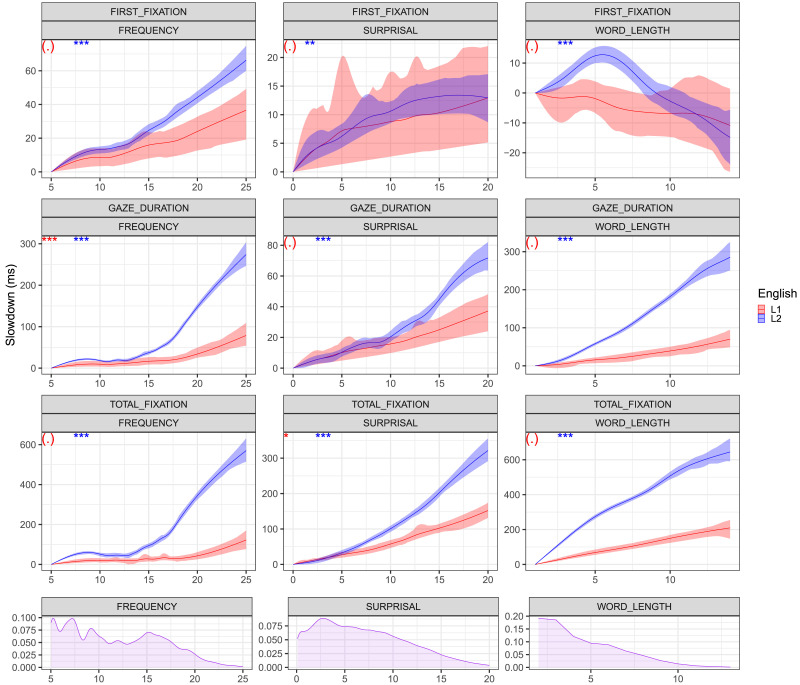
**GAM fits for the relation between benchmark word properties (current word) and raw reading times using the model in Equations 1 and 2**. Upper three rows depict slowdown effects in *ms* as a function of frequency, surprisal and word length for First Fixation, Gaze Duration and Total Fixation, with bootstrapped 95% confidence intervals. Curves are depicted in blue for L1 and in red for L2. At the top left is the significance of the quadratic term when replacing the word property smooth term of the current word with a linear and quadratic terms. ‘***’ *p* < 0.001, ‘**’ *p* < 0.01. ‘*’ *p* < 0.05, ‘(.)’ *p* > 0.05. Bottom row: Density plots for frequency, surprisal and word length values. **Key results**: Superlinear curves for frequency and surprisal in L2 and for frequency Gaze Duration in L1. Stronger superlinearity in L2 than in L1 for both frequency and surprisal. Stronger superlinearity for frequency than for surprisal within both L1 and L2.

Analysis 1 Figure 1 presents GAM fits for the relation between benchmark word properties and raw reading times in L1 and L2. The curves are fitted using mgcv (1.8-31) with cubic regression splines (Wood, 2004). We use the bam function (Wood et al., 2015) with fast REML smoothing parameter estimation. Surprisal curves are fitted with the model:

$$\begin{array}{c} RT∼s\left(\text{surp},bs=“cr”,k=20\right)+s\left({\text{surp}}_{pr},bs=“cr”,k=20\right)\\ \;+te\left(\text{freq},len,bs=“cr”\right)+te\left({\text{freq}}_{pr},{len}_{pr},bs=“cr”\right)\\ \;+s\left(\text{subj},bs=“re”\right)+s\left(\text{subj},\text{surp},bs=“re”\right)\\ \;+te\left(\text{subj},\text{freq},len,bs=“re”\right) \end{array}$$ (1)

and frequency and word length curves are fitted with the model:

$$\begin{array}{c} RT∼s\left(\text{surp},bs=“cr”,k=20\right)+s\left({\text{surp}}_{pr},bs=“cr”,k=20\right)\\ \;+s\left(\text{freq},bs=“cr”,k=20\right)+s\left({\text{freq}}_{pr},bs=“cr”,k=20\right)\\ \;+s\left(len,bs=“cr”\right)+s\left({len}_{pr},bs=“cr”\right)\\ \;+s\left(\text{subj},bs=“re”\right)+s\left(\text{subj},\text{surp},bs=“re”\right)\\ \;+s\left(\text{subj},\text{freq},bs=”re”\right)+s\left(\text{subj},len,bs=“re”\right) \end{array}$$ (2)

where *pr* indicates a property of the previous word (to account for spillover effects; Rayner, 1998).

Terms with *bs* = “*re*” correspond to participant level random effects (not included in the model of Smith & Levy, 2013). We estimate 95% confidence intervals using the bootstrapping method of Smith and Levy (2013). We test for non-linearity of a predictor by replacing its smooth term *s* in the GAM model with a linear and a quadratic terms, and a random slope for the quadratic term, and testing for the significance of the quadratic term, keeping all the other predictors unchanged.

### Effect Size

In Analysis 2 and 3, we compute per participant effect sizes for our three word properties—frequency, surprisal, and length. If we were using ordinary linear regression, the regression coefficients for these word properties would quantify the effect sizes, but with a GAM there is no single regression coefficient for each word property. Instead, for each participant *i* we fit a the GAM model in Equations 1 and 2 without random effects (i.e. without the “re” terms), and quantify the participant’s effect size using the average slowdown for word property *p* as follows:

$${\text{Slowdown}}_{i,p}=\frac{1}{\left|C\right|}\sum_{w\in C}s_{i,p}\left(p\left(w\right)\right)$$ (3)

where *p* ∈ {freq, surp, len} and *s*_*i*,*p*_ is the (potentially nonlinear) partial effect of the word property in participant’s *i* GAM model; we evaluate this partial effect at the property’s value for the word, i.e., *p*(*w*). *C* is the entire corpus. For example, if the shape of participant *i*’s surprisal effect were linear with slope 5 ms per bit, then for a word *w* with a surprisal of 6 bits, *s*_*i*,*p*_(surp(*w*)) would be 30 ms.

**Figure 2.**
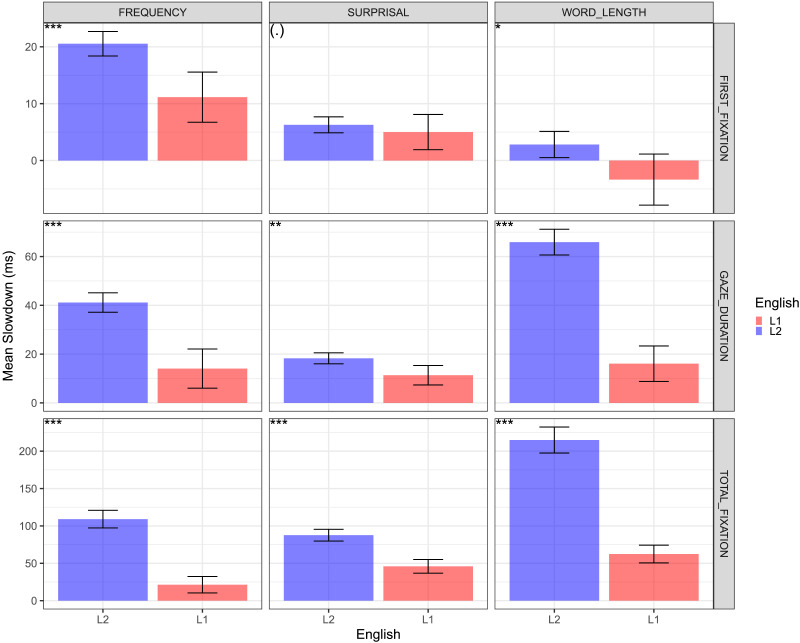
**Mean per subject slowdown effects in ms with 95% confidence intervals based on the GAM model in Equations 1 and 2 fitted separately for each subject (without subject random effects).** The slowdown effect for each subject is calculated using Equation 3. Top left: statistical significance of a *t*-test for the difference between English L1 and English L2. ‘***’ *p* < 0.001, ‘**’ *p* < 0.01. ‘*’ *p* < 0.05, ‘(.)’ *p* > 0.05. **Key results**: Frequency effects are larger in L2 than L1 for all fixation measures. Surprisal effects are larger in L2 than L1 for Gaze Duration and Total Fixation. Differences between L1 and L2 are larger for frequency than for surprisal. While in L1 Total Fixation Duration surprisal is larger than frequency (*p* < 0.001), in L2 the relative importance of frequency remains larger than surprisal (*p* < 0.01).

**Figure 3.**
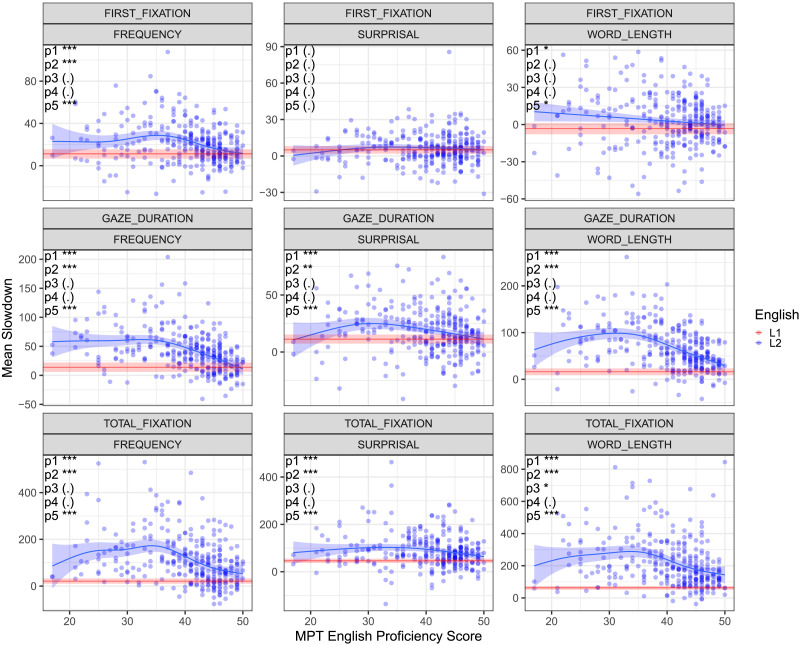
**Slowdown effects associated with benchmark word properties of the current word for raw reading times as a function of English proficiency.** Each blue circle is a single L2 speaker. The *y* axis is the mean slowdown effect for the word property from Equation 3, from the GAM model in Equations 1 and 2, fitted separately for each participant and measure. The *x* axis is the MPT English proficiency score. The blue line is a GAM fit through the L2 slowdown effects *Mean Slowdown* ∼ *s*(*MPT*), and the red line is the mean of the L1 slowdown effects, both with 95% confidence intervals. **Key results**: U shaped relation between proficiency and responsiveness to frequency across all three fixation measures, as well as surprisal for Gaze Duration and Total Fixation. No statistical difference between L2 speakers at the highest proficiency levels and L1 speakers, with the exception of Total Fixation surprisal. Statistical significance of relevant hypothesis tests indicated in top left (see Methods). ‘***’ *p* < 0.001, ‘**’ *p* < 0.01. ‘*’ *p* < 0.05, ‘(.)’ *p* > 0.05.

In Figure 2 we present the mean current word property effects for raw reading times across participants in L1 and L2. Figure 3 depicts current word property effects for raw reading times as a function of English proficiency as measured by the MPT test. A GAM is then fitted to the resulting by-participant L2 slowdown effects to reveal the relationship between participant effect sizes and English proficiency.

### The Lexicon–Context Tradeoff

**Figure 4.**
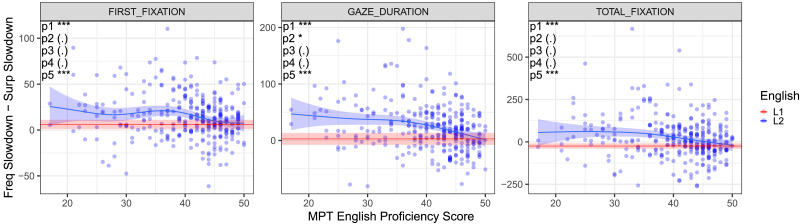
**The frequency–surprisal slowdown effect difference (Equation 4) for current word raw reading times, as a function of English proficiency.** Each blue circle is a single L2 speaker; curves are obtained from the GAM model in Equations 1 and 2 fitted separately for each participant and fixation measure. The *x* axis is the MPT English proficiency score. The blue line is a GAM fit through the L2 values, and the red line is the mean of the L1 values, both with 95% confidence intervals. **Key results**: L1: the importance of frequency compared to surprisal decreases in the transition from First Fixation to Total Fixation. L2: In each measure the importance of frequency compared to surprisal decreases with proficiency. Highly proficient L2 speakers reach a pattern similar to L1 speakers. Statistical significance of relevant hypothesis tests indicated in top left; see also Methods. ‘***’ *p* < 0.001, ‘**’ *p* < 0.01. ‘*’ *p* < 0.05, ‘(.)’ *p* > 0.05.

The difference between the contributions of frequency and surprisal in Figure 4 is computed for each participant *i* as the difference between the respective slowdown effects, i.e.:

$${\text{Diff}}_{i}=\frac{1}{\left|C\right|}\sum_{w\in C}s_{i,\text{freq}}\left(\text{freq}\left(w\right)\right)-s_{i,\text{surp}}\left(\text{surp}\left(w\right)\right)$$ (4)

### The Relationship Between Proficiency and Response to Word Properties

In Analysis 3, we conduct five statistical tests to answer key questions about the shape of the relationship between MPT-measured English proficiency and the sensitivity of eye movement measures to word frequency, length, and surprisal, as detailed below. Each test involves fitting a multiple regression model and testing the significance of the model term corresponding to the question. For these analyses, native English speakers are assigned the maximum possible MPT score.

First, are the effects of L2 English proficiency non-linear? To answer this question we conducted the following tests:

$$\begin{array}{cc} m1:\text{response}∼s\left(MPT\right) & \left(\text{testing}\;\text{the}\;\text{smooth}\;\text{term}\right)\\ m2:\text{response}∼MPT+{MPT}^{2} & \left(\text{testing}\;\text{the}\;\text{quadratic}\;\text{term}\right) \end{array}$$

Second, are the most proficient L2 speakers’ effect sizes indistinguishable from those of native speakers? To answer this question we included a 0/1 predictor variable indicating whether the participant was an English L1 reader, and tested the significance of this term for the following two models:

$$\begin{array}{cc} m3:\text{response}∼\text{English}+MPT & \left(\text{including}\;\text{only}\;\text{participants}\;\text{with}\;\text{above}‐\text{median}\;\text{MPT}\right)\\ m4:\text{response}∼\text{English}+MPT+{MPT}^{2} & \left(\text{including}\;\text{all}\;\text{participants}\right) \end{array}$$

Third, among above-average L2 readers, do the effect sizes increase with decreasing proficiency? To answer this question we tested the significance of the linear *MPT* term in the following model applied to participants with above-median MPT score:

m5:response∼MPT

In Figures 3 and 4, the statistical significance levels *p*1 − *p*5 correspond to these tests for models *m*1 − *m*5 respectively.

### Preregistration

The analyses in this paper were pre-registered at http://osf.io/azrh3 for v1 of CELER which comprises 182 participants (Part 1). The remaining data from 183 participants (Part 2) was held out for confirmatory analyses. In Figures S13–S20 of the Supplemental Materials, we provide the analyses results separately for Part 1 and Part 2. The results are highly consistent across the two parts, as well as with all 365 participants.

## RESULTS

### Analysis 1: Functional Form

Our first analysis characterizes the functional form of the relation between reading times and benchmark word properties in English L1 and L2. In this and the following two analysis we focus on the effects of frequency and predictability, while also presenting word length results for additional reference. Following Howes and Solomon (1951) and others, models of eye movements in reading generally assume a linear relationship between log-frequency and reading times (Engbert et al., 2005; Reichle et al., 2003). However, recent work has suggested that this relation might be superlinear in the low frequency range (Kuperman & Van Dyke, 2013; White et al., 2018; Wotschack & Kliegl, 2013). This result is in line with word recognition studies which yielded a superlinear relation for frequency in both L1 and L2, with a stronger superlinearity for L2 compared to L1 (Diependaele et al., 2013; Lemhöfer et al., 2008). For predictability, Smith and Levy (2013), Goodkind and Bicknell (2018), and Wilcox et al. (2020) found a linear relationship between reading times in L1 and predictability as measured by corpus based surprisal.

Despite these advances, the functional form of both frequency and predictability effects in L2 reading has not been characterized to date. Prior work that examined frequency effects in L1 and L2 assumed a linear effect of log frequency on reading times (Cop, Keuleers, et al., 2015; Mor & Prior, 2022; Whitford & Titone, 2012). Whitford and Titone (2017) and Mor and Prior (2022) assumed a linear effect of (untransformed) predictability. Here we relax this linearity assumption, using nonparametric statistical methods to find the functional form best supported by the data.

In this and subsequent analyses we quantify word predictability as corpus based surprisal (Hale, 2001; Levy, 2008) using the GPT2 language model (Radford et al., 2019) whose surprisal estimates were shown to correlate well with reading times in English L1 (Heilbron et al., 2022; Wilcox et al., 2020). We use three standard fixation measures:

1. First Fixation: the duration of the first fixation on a word.
2. Gaze Duration: the time from first entering the word to first leaving it.
3. Total Fixation: the sum of all fixations on a word.

These three measures stand in a temporally monotonic inclusion relationship: the earliest time period of a word’s Gaze Duration is its First Fixation, and the earliest time period of a word’s Total Fixation time is its Gaze Duration. Hence measures 1–3 capture successively later stages of language processing (Inhoff, 1984; Liversedge & Findlay, 2000; Rayner, 1998).

We estimate the functional relationship between benchmark word properties and fixation times using General Additive Models (GAMs). The model, specified in Equations 1 and 2, predicts reading times from the frequency, surprisal and word length of the current and the previous words. We fit this model separately for the L1 and L2 groups, and each of our three fixation measures. We test for superlinearity of a predictor by replacing its smooth term in the GAM model with linear and quadratic terms, and testing for the significance of the quadratic term.

Figure 1 depicts the resulting curves for the current word. Figure S1 in the Supplemental Materials further presents spillover effects from the previous word. For L1 frequency, we observe a linear relation for First Fixation while also obtaining evidence for superlinearity in the low frequency range for Gaze Duration as suggested in (Kuperman & Van Dyke, 2013; White et al., 2018; Wotschack & Kliegl, 2013). Visual inspection of Total Fixation suggests a similar trend, although the superlinearity is not statistically significant. L1 surprisal curves are linear, largely replicating prior work, with the exception of weakly superlinear curve for Total Fixation. For L2, however, frequency effects are superlinear for all three measures and surprisal effects are superlinear for Gaze Duration and Total Fixation. These outcomes do not support the linearity assumption previously taken in the literature when analyzing frequency and surprisal effects in L2 reading data. Figure S2 of the Supplemental Materials shows that these functional forms are preserved after reading times normalization. Figures S3 and S4 in the Supplemental Materials further break down the L2 current word results by native language, indicating that the functional form results hold across the languages in our sample. Figure S11 further provides mean fixation durations by L1. Overall, we observe more superlinearity in L2 compared to L1 for both frequency and surprisal, and stronger superlinearity for frequency than surprisal within L1 and L2. We further note substantial differences in the magnitude of the L1 and L2 effects, with larger discrepancies between L1 and L2 for frequency than for surprisal. We examine these differences further in Analysis 2.

The superlinearity of frequency effects can be interpreted with respect to lexical knowledge, which is an important factor in reading comprehension ability (Perfetti, 2007). It is possible that words which are not in the participant’s lexicon will introduce a substantial overhead to their expected processing time from a linear function, resulting in a superlinear response. As the probability of any lexical item being unknown to the speaker is higher in L2 than in L1, the superlinearity is stronger for L2. Similarly, the superlinearity in L2 surprisal is likely to be related to the limited ability of this population to perform meaningful contextual integration. A key avenue for future work will be developing a formal model for these curves, where a key challenge will be accounting for the larger L1–L2 discrepancies for frequency as compared to surprisal.

### Analysis 2: Magnitude

In our second analysis we use a summary view of Analysis 1, to quantify and compare the overall magnitudes of benchmark word property effects in L1 and L2. Whitford and Titone (2012), Cop, Keuleers, et al. (2015) and Mor and Prior (2022) observed larger frequency effects in L2 compared to L1. This outcome is consistent with studies which obtained larger L2 than L1 frequency effects in single word recognition and production tasks, including lexical decision (Duyck et al., 2008; Van Wijnendaele & Brysbaert, 2002), progressive demasking (Diependaele et al., 2013; Lemhöfer et al., 2008), word naming (de Groot et al., 2002) and picture naming (Gollan et al., 2008). Whitford and Titone (2017) found no evidence for L1 versus L2 differences for predictability effects, while Mor and Prior (2022) found larger L2 than L1 effects for Total Fixation Duration. As Analysis 1 suggests superlinearity in many of the relevant effects, here we examine their magnitude without assuming linear effect shapes.

To calculate effect magnitude, we fit the GAM model in Equations 1 and 2 for each subject (without the by-subject random effects), and calculate the subject’s mean word property slowdown effect across all the words in the corpus using Equation 3. Figure 2 depicts the average slowdown effect across subjects for L1 and L2. Consistent with Whitford and Titone (2012), Cop, Keuleers, et al. (2015) and Mor and Prior (2022), the effect of frequency is larger in L2 than in L1 for all three fixation measures. Differently from Whitford and Titone (2017) and in line with Mor and Prior (2022), we find that the effect of surprisal in L2 is larger than L1 for Gaze Duration and Total Fixation. As observed in Analysis 1, L1 versus L2 differences for surprisal are considerably smaller than for frequency. Importantly, we see that in the transition from Gaze Duration to Total Fixation, L1 speakers end up with a substantially larger effect for surprisal than for frequency, while in L2 frequency effects remain larger than surprisal. We further note that the response to surprisal is delayed compared to frequency in both L1 and L2. Figure S5 in the Supplemental Materials depicts this analysis for speed normalized fixation times, where we observe that larger L2 than L1 effects persist for frequency, but not for surprisal. Figures S6 and S7 break down the analysis by native language, and suggest that the results largely hold across different native languages. We also note potential magnitude differences between native languages for frequency and word length, which are also apparent in S3 and S4 of Analysis 1, whose investigation we leave for future work.

Taken together, the results of Analyses 1 and 2 exhibit a marked difference in the dynamics of the frequency and surprisal predictors within and across the L1 and L2 groups. The differences are consistent with an interpretation that frequency and surprisal tap into different cognitive processing mechanisms, where frequency is associated with lexical processing and surprisal with contextual processing, with the former generally preceding the latter (Staub, 2011). This interpretation is also consistent with the observation that reading times in L2 differ from L1 primarily in larger frequency effects which are more pronounced than surprisal even at the latest stages of processing, suggesting a larger role for lexical processing compared to contextual processing as determinants of processing load in non-native language comprehension.

### Analysis 3: Interaction with L2 Proficiency

Our final analysis examines how responsiveness to benchmark word properties depends English L2 proficiency. In prior work, Whitford and Titone (2012) and Cop, Keuleers, et al. (2015) have found that the magnitude of L2 frequency effects in reading is inversely related to self reported L2 exposure. However, Cop, Keuleers, et al. (2015) did not find such an interaction for linguistic proficiency, which was approximated in their study using the LexTALE English vocabulary test (Lemhöfer & Broersma, 2012). In the word recognition domain, Diependaele et al. (2013) did find a frequency—LexTALE proficiency interaction both in L1 and L2 speakers of English. Mor and Prior (2022) examined the relation between word predictability and proficiency, approximated from a combination of the Shipley vocabulary test (Shipley, 1946) and the TOWRE reading fluency test (Torgesen et al., 1999), and found no evidence for an interaction between the two.

Differently from previous approaches in the literature which use linear modelling (Cop, Keuleers, et al., 2015; Diependaele et al., 2013; Mor & Prior, 2022; Whitford & Titone, 2012), here we do not assume linearity and characterize the functional form of this interaction. To this end, as in Analysis 2, we fit the GAM model in Equations 1 and 2 separately for each participant (without the by-subject random effects), and compute an average word property slowdown effect for each participant using Equation 3. We then fit a GAM through the resulting participant slowdown effects as a function of language proficiency, as measured by the listening comprehension and grammar sections of the Michigan Placement Test (MPT).

Figure 3 presents the resulting curves against the mean slowdown effect of the L1 group. We observe that the linear interaction approach in the literature underfits the data. For all three measures, the frequency effect sizes are U-shaped; effect sizes initially increase with decreasing proficiency, but the slope of this relationship diminishes or reverses with even lower proficiency. The highest proficiency L2 readers’ effect size is statistically indistinguishable from native readers. These visually apparent results are statistically confirmed in five hypothesis tests; see Methods. Similar results are observed for surprisal in Gaze Duration and Total Fixation. Figure S8 of the Supplemental Materials shows that these results are preserved for frequency when accounting for reading speed. Figure S21 of the Supplemental Materials further shows that the results largely hold when replacing the MPT with the percentage of correctly answered reading comprehension questions during the eye-tracking experiment.

This outcome opens an intriguing question on the role of text difficulty in the effect of word properties on reading times. One possibility is that high discrepancy between text difficulty and proficiency results in more text skimming, as evidenced by the decrease in the mean fixation durations in the low proficiency range in Figure S12 in the Supplemental Material. Prior research suggests that mindless reading weakens the response to word properties (Reichle et al., 2010). When low proficiency participants read challenging newswire text, they might be engaging in skimming-like behavior more than higher proficiency readers, leading to faster reading and smaller word property effects. Alternatively, the non-linear modulation of proficiency on word property effects could also be invariant to the difficulty level of the text. We leave this question for future research.

Finally, in Figure 4 we fit individual participant models identical to those in Figure 3, and then depict the mean difference between the slowdown effects associated with frequency and surprisal across all the words in the corpus for each participant using Equation 4. Consistent with the previous analyses, for L1 readers the importance of surprisal compared to frequency increases from First Fixation to Total Fixation. L2 readers are able to rely increasingly more on facilitation from context-based prediction, with the most proficient L2 readers reaching an L1-like pattern in all three fixation measures. Figure S9 of the Supplemental Materials presents similar results with normalized reading times. Figure S22 of the Supplemental Materials shows that the results hold when using reading comprehension scores in place of the MPT.

## DISCUSSION

Our analyses yield the following primary results.

1. **Functional Form**: In L1, we find that fixation times are linear in surprisal, and weakly superlinear in frequency. In L2, the relation between reading times and frequency, as well as surprisal, is *superlinear*. Overall, we observe stronger superlinearity for frequency than surprisal across L1 and L2, and stronger superlinearity for L2 compared to L1 across frequency and surprisal.
2. **Magnitude**: Both frequency and surprisal effects are *larger* in L2 compared to L1. Further, differences between L1 and L2 are larger for frequency than for surprisal. Our analysis also yields differences in the time course of the response to frequency and surprisal, both within and across the L1 and L2 groups. In particular, differently from L1 where the relative importance of frequency is smaller the later the stage of processing captured by a fixation measures, in L2 frequency effects remain larger than surprisal across all fixation measures.
3. **Interaction with L2 proficiency**: The modulation of L2 proficiency on frequency and surprisal effects is non-linear; they increase as language proficiency decreases, then saturate and possibly decrease in the low proficiency range. The most proficient L2 speakers exhibit frequency and surprisal effects similar to those of L1 speakers.

These results suggest that although L2 reading is qualitatively similar to L1, it also differs from L1 in the dominance of frequency effects over surprisal effects. This outcome is consistent with the theoretical account of rational processing, and suggests a key difference between L2 and L1 in what we refer to as a *lexicon–context tradeoff*: context-based prediction plays a less central role in affecting eye movements in reading for L2 speakers than for L1 speakers, but this is modulated by L2 proficiency, with the most proficient L2 speakers approaching a fully L1-like pattern. These results suggest that with language learning comes a gradual shift in the online dynamics of language processing, away from lexical processing and towards contextual processing.

Our analyses speak to a number of fundamental questions in language processing and language learning. First, our results are largely consistent with “lexical entrenchment” (Diependaele et al., 2013) and “weaker links” (Gollan et al., 2008) accounts, which posit that linguistic knowledge is inversely related to frequency effects. However, tracing how benchmark word property effects depend on language proficiency reveals that this trajectory is not monotonic.

Next, this work is pertinent to the general interpretation of frequency and surprisal effects in reading. While many studies found both frequency and surprisal effects in reading, their co-existence poses a theoretical challenge. Since log-frequency is formally simply unigram surprisal, it might be expected to be subsumed by surprisal. It is currently an open question whether frequency and surprisal encode different mechanisms, and recent experimental results suggest that surprisal may indeed subsume frequency (Shain, 2019). Our results do not support this view; they not only reinforce previous accounts of frequency effects being present above and beyond surprisal, but also suggest different time course dynamics and different effects of frequency and surprisal within and across L1 and L2. This suggests that common computational constraints apply to both L1 and L2 readers, but are more severe for L2 readers, the more so the lower the reader’s proficiency. Overall, our results are consistent with the interpretation that frequency taps into lexical processing while surprisal is associated with contextual processing.

Finally, our results indicate that the differences between L1 and L2 are substantially more pronounced with respect to frequency than surprisal. This is reflected both in the larger gaps between L1 and L2 frequency effects, as well as in the larger degree of superlinearity in the relation between reading times and frequency as compared to surprisal. Further, relative to surprisal, frequency effects play a more dominant role in L2 compared to L1. Given these results, and our association of frequency with lexical processing and surprisal with contextual processing, L1 speakers are able to rely more heavily on contextual facilitation. The process of L2 learning involves a gradual shift in a lexicon-context tradeoff, with diminishing importance of lexical processing to the overall process of language comprehension. The most proficient L2 speakers exhibit a tradeoff indistinguishable from L1.

A potential caveat to this interpretation of our results is the possibility that language model based surprisals are a less accurate estimate of L2 subjective probabilities than of L1, and a worse estimator for less proficient L2 speakers. The simplest account one could propose might be that the learning and expectation-deployment mechanisms for L2 and L1 speakers are the same, but L2 readers are disadvantaged by a smaller linguistic sample size than L1 readers (amount of exposure to the language) for learning expectations. Smaller sample size means more variability, and thus more potential discrepancy between L2 reader expectations and the properties of the read texts, especially for rare events that are unlikely to have occurred often in a reader’s experience. These discrepancies could thus lead to greater reading-time penalties for rare and surprising words, potentially yielding both non-linear effect shapes (as seen in Analysis 1) and larger effect sizes (as seen in Analysis 2).

However, based on initial computational simulations we have conducted to investigate these issues, which we report in Appendix A, we believe that it is unlikely that an account based solely on the size of the learning sample would satisfactorily explain the patterns we see in our data: apparent nonlinearities resulting from sample size effects are minimal for word frequency, and subjective expectations learned from small samples do not substantially magnify apparent effect sizes for word frequency and surprisal estimates that are based on a larger training corpus (in many cases, they lead to reduced effect sizes). Why the quantitative effect shape and size effects are seen in our data is therefore, we believe, an important theoretical and empirical question. In the future, machine learning techniques for estimating L2 lexica and context-based word predictions could be used to develop and evaluate computationally implemented hypotheses.

An additional limitation of this work is that CELER comprises out-of-context single sentences. While single sentence corpora have been widely used in psycholinguistics, conclusions drawn from such datasets regarding word predictability cannot take into account extra-sentential context. Is is currently an open question whether our conclusions will generalize to contextualized reading of full passages. We plan to examine this question in future work.

Ultimately, an important challenge in cognitive science is the formulation of computational models which predict eye movements in reading based on fundamental principles of language processing and acquisition. To accurately capture the full scope of human reading behavior, such models will have to account for the variability in the linguistic knowledge and experience of readers, and reproduce their effects on fixation times. Our study takes a step forward in delineating the empirical landscape that will guide the development of such models.

## ACKNOWLEDGMENTS

This work was supported NSF STC award CCF-1231216, MIT–IBM AI research lab, the MIT Quest for Intelligence, NSF grant IIS-1815529, BCS-2121074 and ISF grant 2070358.

## DATA AVAILABILITY STATEMENT

Code for this paper is available here: https://github.com/lacclab/traces-of-ling-knowledge. Data is available here: https://github.com/berzak/celer.

## Notes

^1^ This can be mathematically justified. Surprisal and negative log-frequency are convex functions, Jensen’s Inequality states that for a function *f* that is nonlinear on the set of values that can be taken by a random variable *x*, *E*[*f*(*x*)] ≥ *f*(*E*[*x*]). In our setting, *x* is the subjective frequency or conditional probability of a given word, *E*[*x*] is the population-average conditional probability (which by hypothesis is well estimated by the large reference corpus), and Jensen’s inequality tells us that the variability of *x* will magnify word frequency and surprisal effects. The less linguistic experience of the reader, the more variable their word log-frequency and surprisal estimates will be, and thus the greater the magnification of the effect.^2^ When generating the reading-time data, we exclude tokens from the reading corpus that involve zero-count events in any of the reference, small, or large corpora.

## Figures and Tables

**Figure A1.**
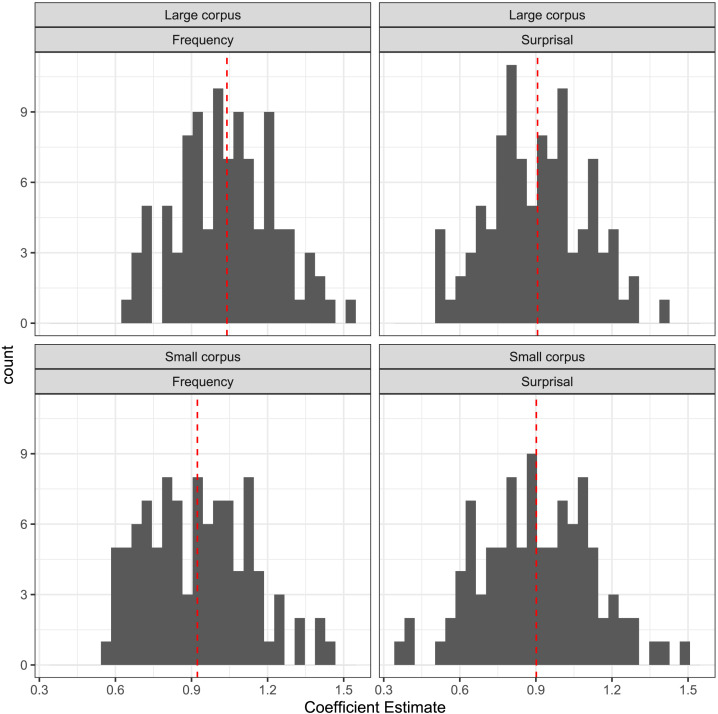
**Histograms of coefficient estimates for word frequency and surprisal effects on continuous eye movement measure based on simulated corpora of varying size (see main text for details).** Red dashed lines indicate mean estimate. The mean estimated frequency effect is very slightly smaller (closer to zero) when based on larger corpora than on smaller corpora; the mean estimated surprisal effects are indistinguishable.

**Figure A2.**
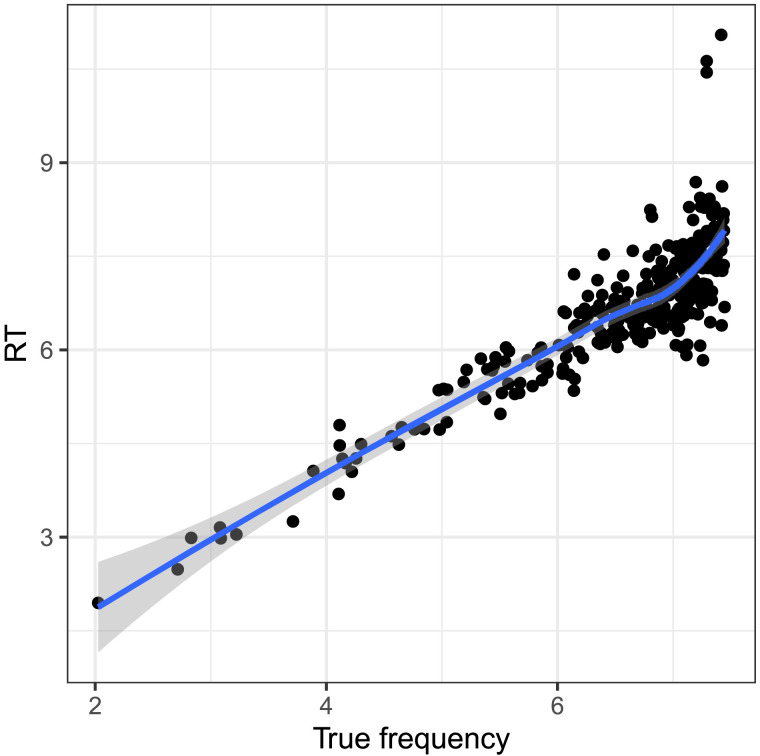
**Effect of less linguistic exposure as the basis of a word frequency.** The nonlinearity induced by sampling error due to the small sample size assumed for the non-native reader is minimal, and confined to the far low-frequency tail of the graph.

## APPENDIX A: SIMULATION-BASED ANALYSIS OF THE EFFECT OF QUANTITY OF LINGUISTIC EXPOSURE ON ESTIMATED WORD FREQUENCY AND SURPRISAL EFFECTS

### Effect Size

One question raised in the discussion of our results is whether the difference in word frequency and surprisal effect sizes for L1 and L2 populations found in Analysis 2 might derive simply from differences in the amount of speakers’ linguistic experience. Theoretically this is possible: intuitively, the less linguistic experience from which a speaker’s subjective word frequencies and surprisals are derived, the more variable they will be from speaker to speaker and the more they will differ from estimates derived from a large reference corpus used by a researcher, inflating the effect sizes of frequency and surprisal as estimated from the reference corpus.^1^ However, it is not clear whether this effect of variability on effect size would be substantial enough to account for the L1/L2 differences we see in our data. To get a handle on this question, we turn to a simulation-based approach.

Our simulations have the following structure. We start with the Wikitext-2 dataset (Merity et al., 2016) and use relative frequency estimation to estimate unigram and bigram models of English (treating the corpus as a loop to avoid issues with beginning and end of corpus). We then use the bigram model to sample a *reference* corpus (simulating the dataset used by a researcher to train a language model), a *large* corpus (simulating an L1 speaker’s linguistic experience), a *small* corpus (simulating an L2 speaker’s linguistic experience), and a *reading* corpus (simulating the texts on which eye movement measures during reading are collected from L1 and L2 speakers). Bigram and unigram models are relative-frequency estimated for the reference, large, and small corpora, and reading-time data are generated stochastically for the reading corpus with word surprisal and negative log-frequency effects from the (i) small; and (ii) large; unigram and bigram models respectively.^2^ Linear regression is then used to estimate the effects of *reference* corpus log-frequency and surprisal on the resulting two reading-time datasets, and we investigated the distributions of these word effects. The true linear model coefficients relating word surprisal and negative log-frequency (from small or large corpora) to the reading-time measure are *β*_Surprisal_ = *β*_Frequency_ = 1, and residual error is taken to be normally distributed with standard deviation 5.

Histograms of these coefficient estimates are shown in Figure A1. For surprisal (both corpus sizes) and small-corpus word frequency, the mean coefficient estimates are around 0.9, smaller than the true underlying coefficient values of 1, due to the effect of measurement error from using the reference corpus-derived predictor values instead of the true subjective values (from the large/small corpora). However, for large-corpus word frequency effects, the mean coefficient estimate is right around the true underlying coefficient value of 1.

We conclude from this simulation that a pure ”smaller sample size” account is unlikely to satisfactorily explain the differing effect sizes observed in our datasets: at least as far as our simulation suggests, apparent frequency effects would be *larger* for L1 speakers than for L2 speakers, due to the more severe measurement error for estimation of L2 word frequencies.

### Effect Shape

In Analysis 1 we found clear evidence of superlinearity in word frequency and surprisal effects for L2 speakers, whereas these effects were much closer to linear in L1 speakers. As with the effect size question addressed earlier in this Appendix, an important question here is whether this superlinearity might be a straightforward consequence of the amount of linguistic experience from which a reader derives subjective word frequency and surprisal. This argument would proceed as follows: for readers with less experience (smaller sample size from which learning occurs) like L2 readers, log-frequency and surprisal estimates will be more variable than for readers with more experience like L1 readers. Due to the log transform relating frequency and word probability to reading-time measures, this will translate into more variability in effect sizes in low-probability regions, thus affecting lower-frequency and higher-surprisal words more (and for surprisal affecting rarer contexts more). So, even if true underlying subjective log-frequency and surprisal accounts are linear, it could look like superlinearity in “true” L1-like log-frequency and surprisal (estimated from more data).

However, we have investigated this effect through simulations, and to our surprise it turns out to be very small. Our simulations focused on word frequency and proceeded as follows. We collect a word frequency distribution from Wikitext-2, and then downsample the distribution by a factor of 200. We then assume that word frequency effects are linear in the downsampled word frequencies, generate random word-average RTs, and analyze the shape of the “true” (Wikitext-2) log-frequency effect on RTs using GAMs and quadratic regression. In some samples the shape can turn out to be superlinear, but the superlinearity is minimal and confined to only the very rarest words—it is the slight upward bend in the rightmost part of the graph in Figure A2, and rarely reaches statistical significance at *p* < 0.05 in simulations.

We conclude from this that a pure “smaller sample size” account is unlikely to be enough to fully explain the superlinearity effects we observed—especially because our superlinearity is at least as pronounced in log-frequency as in surprisal, whereas on a pure sample-size account we would expect, if anything, the reverse (since the downsampling is even more extreme when it is context-specific). We believe that additional mechanisms beyond raw quantity of linguistic experience will need to be appealed to in order to explain the superlinearities seen in L2 readers’ word frequency and surprisal effects.
