# Supplementary material for: Eye Movement Traces of Linguistic Knowledge in Native and Non-Native Reading
Source: Open Mind (Camb). 2023 Jun 5;7:179–96. doi: 10.1162/opmi_a_00084 (PMC10320821; doi:10.1162/opmi_a_00084)
Supplement: Supplementary file 1 [file opmi-07-179-s001.pdf]

Eye Movement Traces of Linguistic Knowledge in Native and Non-Native Reading

Supplemental Material

May 24, 2023

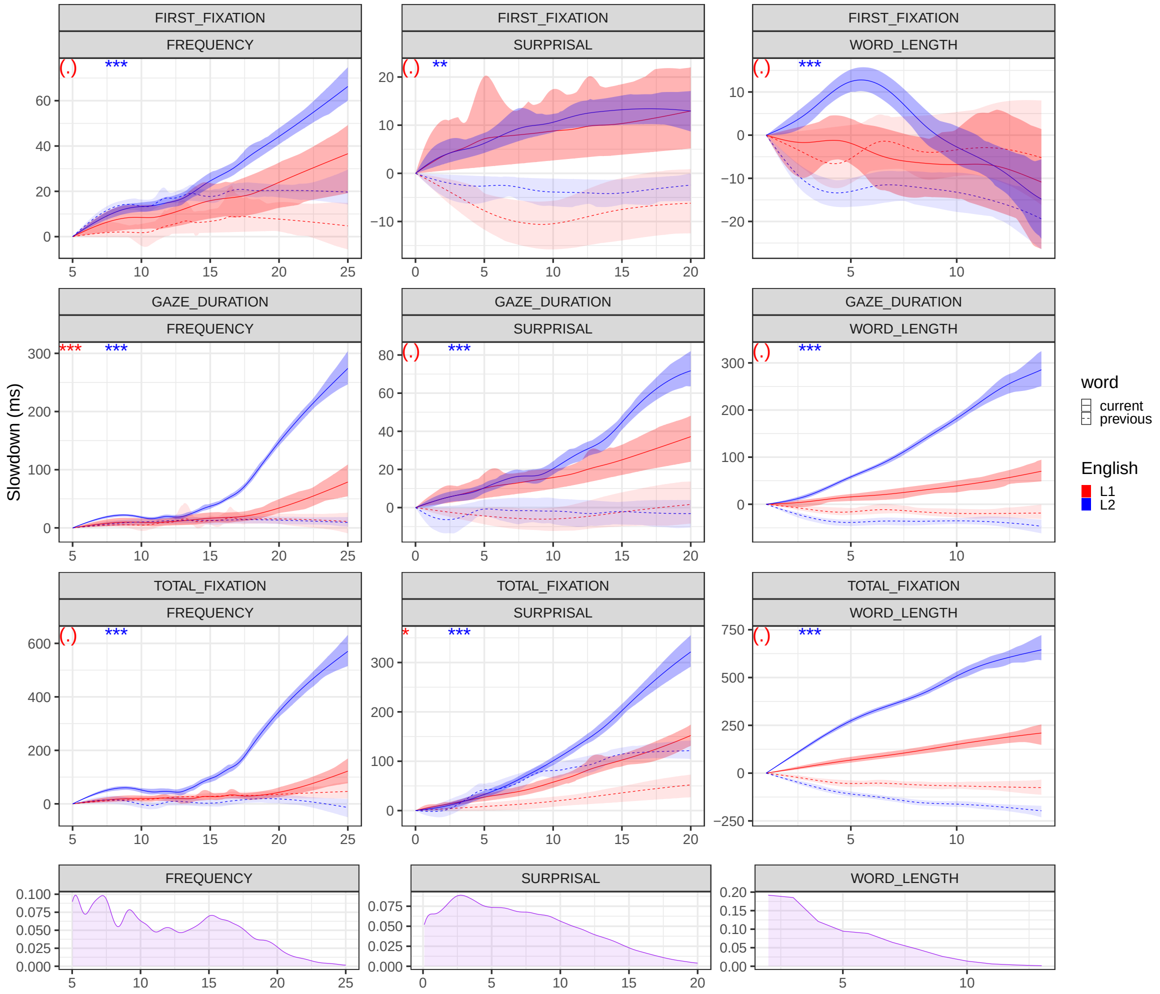

Figure S1: GAM fits for the relation between word properties and **raw reading times** using the model specified in Method. Upper three rows depict slowdown effects in *ms* as a function of frequency, surprisal and word length for First Fixation, Gaze Duration and Total Fixation, with bootstrapped 95% confidence intervals. Continuous and dashed lines correspond to the word property of the current and previous word respectively. Curves are depicted in blue for L1 and in red for L2. At the top right is the significance of the quadratic term when replacing the word property smooth term of the current word with a linear and quadratic terms. '***' $p < 0.001$, '**' $p < 0.01$. '*' $p < 0.05$, '(.)' $p > 0.05$. Bottom row: Density plots for frequency, surprisal and word length values.

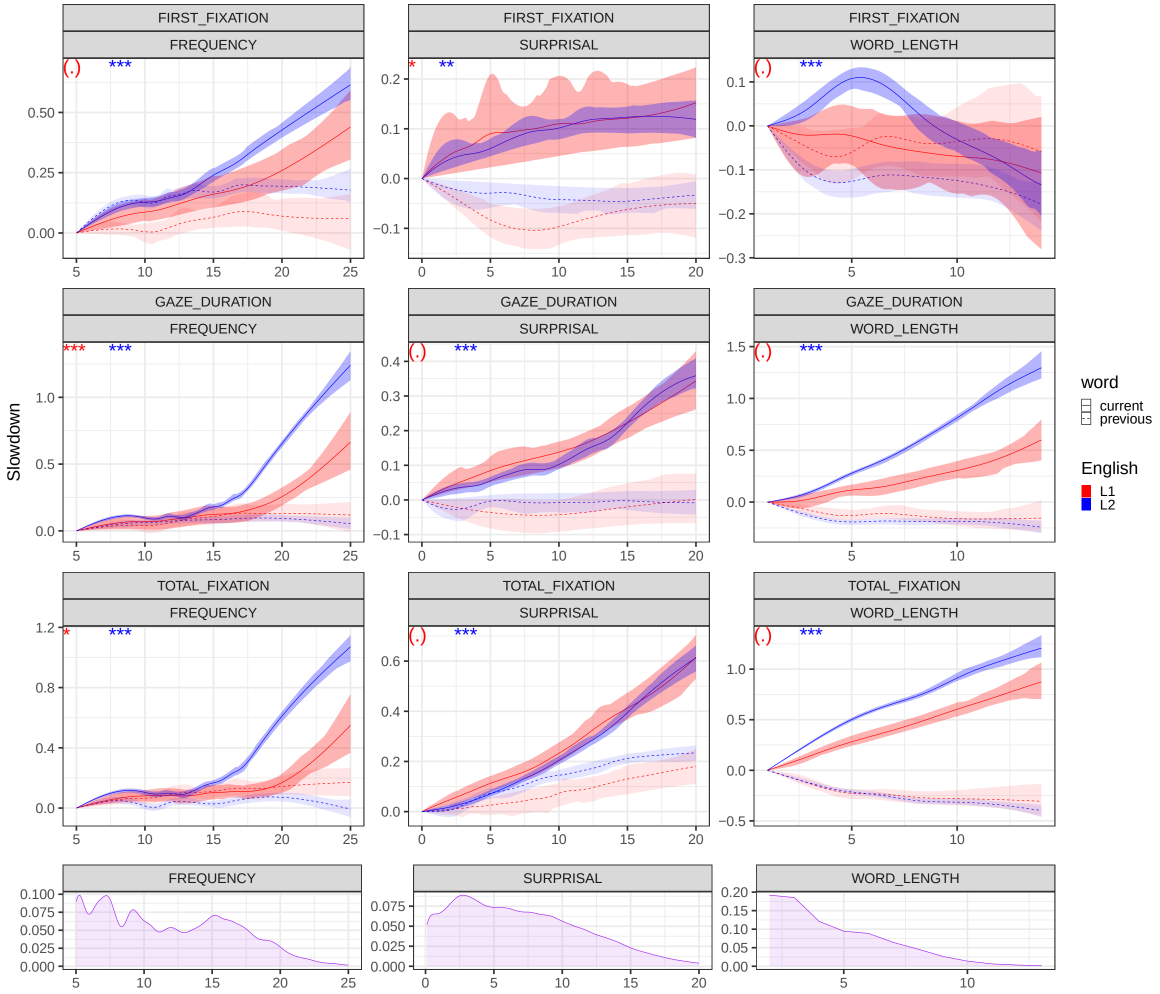

Figure S2: GAM fits for the relation between word properties and **Z normalized reading times** using the model specified in Method. Upper three rows depict slowdown effects as a function of frequency, surprisal and word length for First Fixation, Gaze Duration and Total Fixation, with bootstrapped 95% confidence intervals. Continuous and dashed lines correspond to the word property of the current and previous word respectively. Curves are depicted in blue for L1 and in red for L2. At the top left is the significance of the quadratic term when replacing the word property smooth term of the current word with a linear and quadratic terms. '***' $p < 0.001$, '**' $p < 0.01$. '*' $p < 0.05$, '(.)' $p > 0.05$. Bottom row: Density plots for frequency, surprisal and word length values.

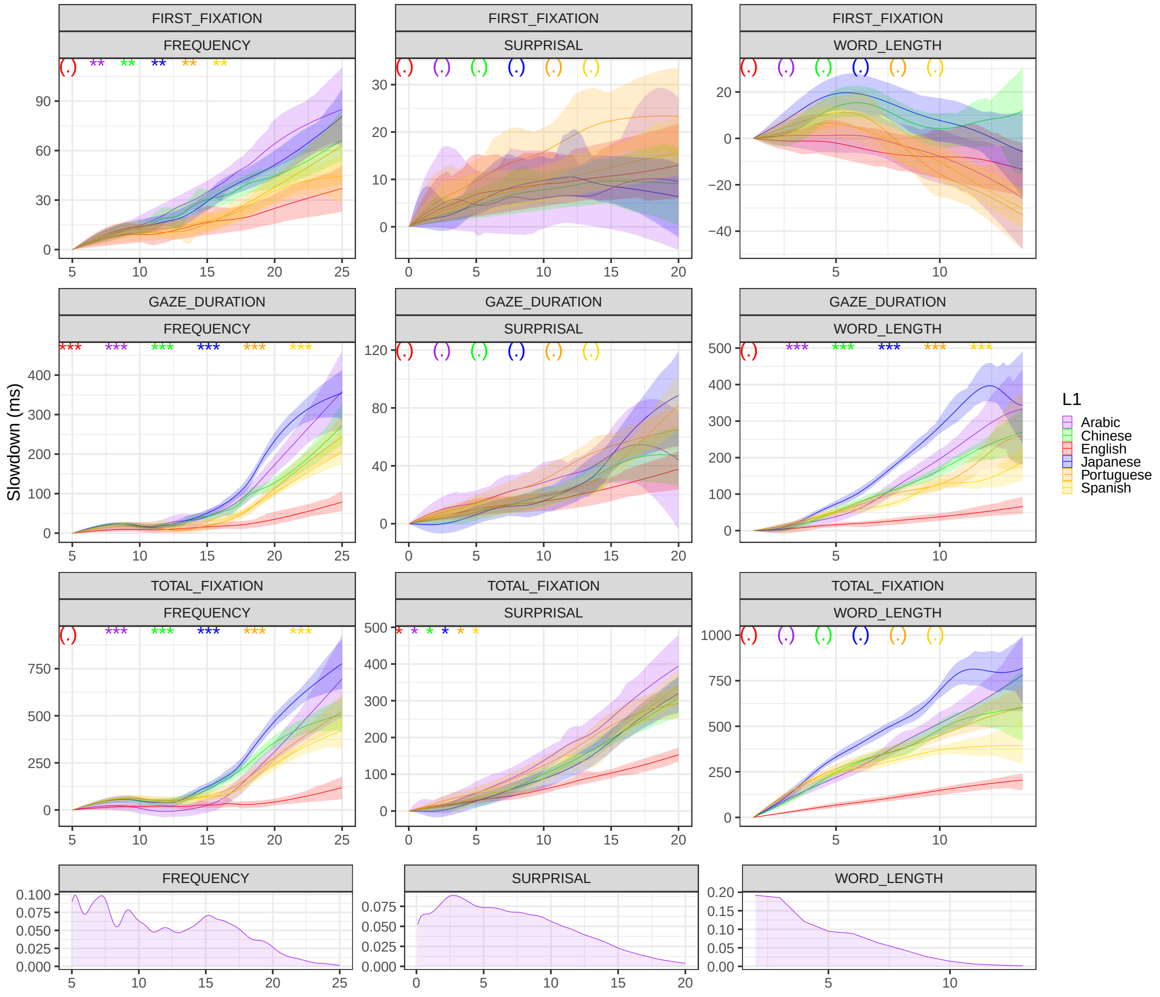

Figure S3: GAM fits for the relation between word properties (current word) and **raw reading times** by participant L1 using the model specified in Method. Upper three rows depict slowdown effects in *ms* as a function of frequency, surprisal and word length for First Fixation, Gaze Duration and Total Fixation, with bootstrapped 95% confidence intervals. At the top left is the significance of the quadratic term when replacing the word property smooth term of the current word with a linear and quadratic terms. '***' $p < 0.001$, '**' $p < 0.01$. '*' $p < 0.05$, '(.)' $p > 0.05$. Bottom row: Density plots for frequency, surprisal and word length values.

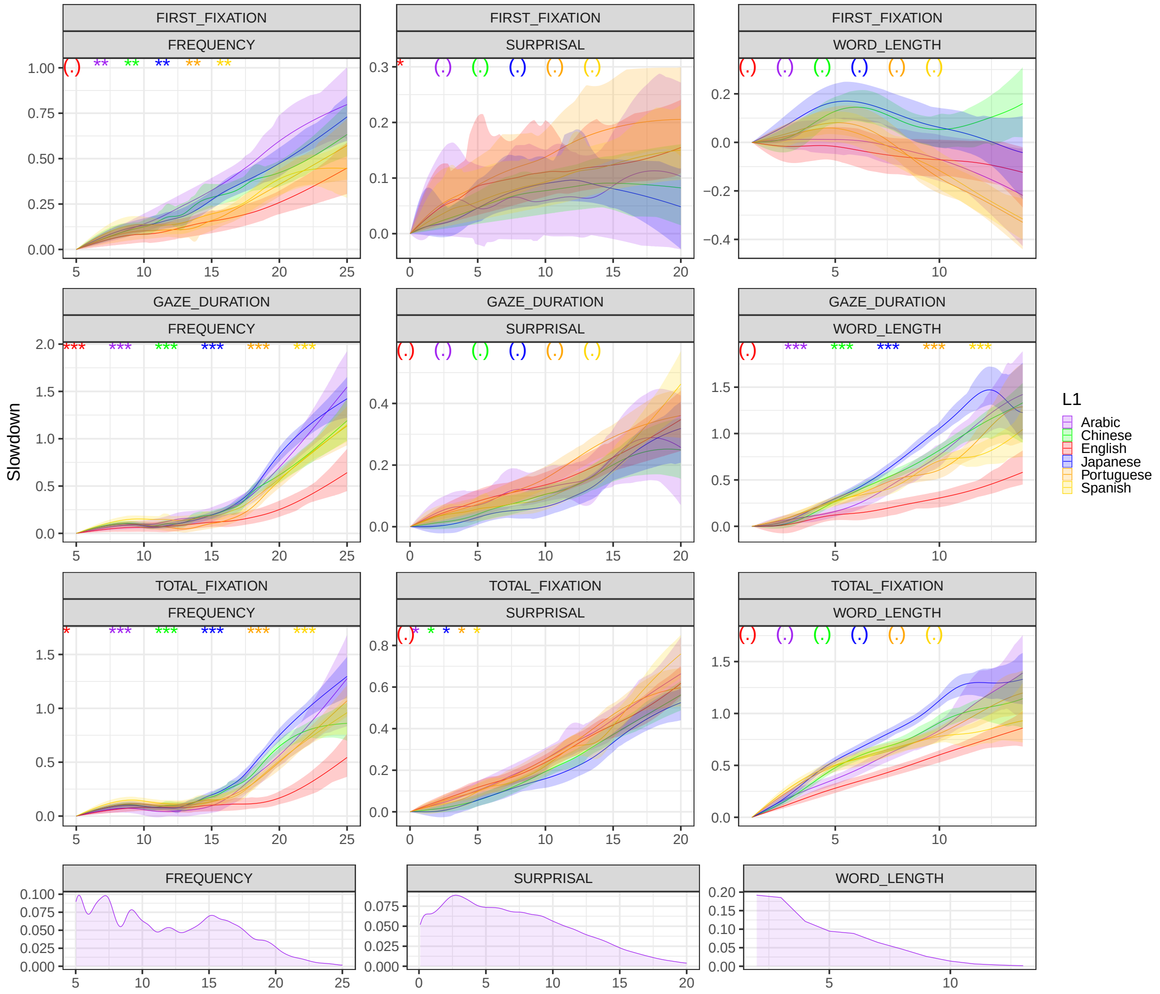

Figure S4: GAM fits for the relation between word properties (current word) and **Z normalized reading times** by participant L1 using the model specified in Method. Upper three rows depict slowdown effects as a function of frequency, surprisal and word length for First Fixation, Gaze Duration and Total Fixation, with bootstrapped 95% confidence intervals. Continuous and dashed lines correspond to the word property of the current and previous word respectively. Curves are depicted in blue for L1 and in red for L2. At the top left is the significance of the quadratic term when replacing the word property smooth term of the current word with a linear and quadratic terms. '***' $p < 0.001$, '**' $p < 0.01$. '*' $p < 0.05$, '(.)' $p > 0.05$. Bottom row: Density plots for frequency, surprisal and word length values.

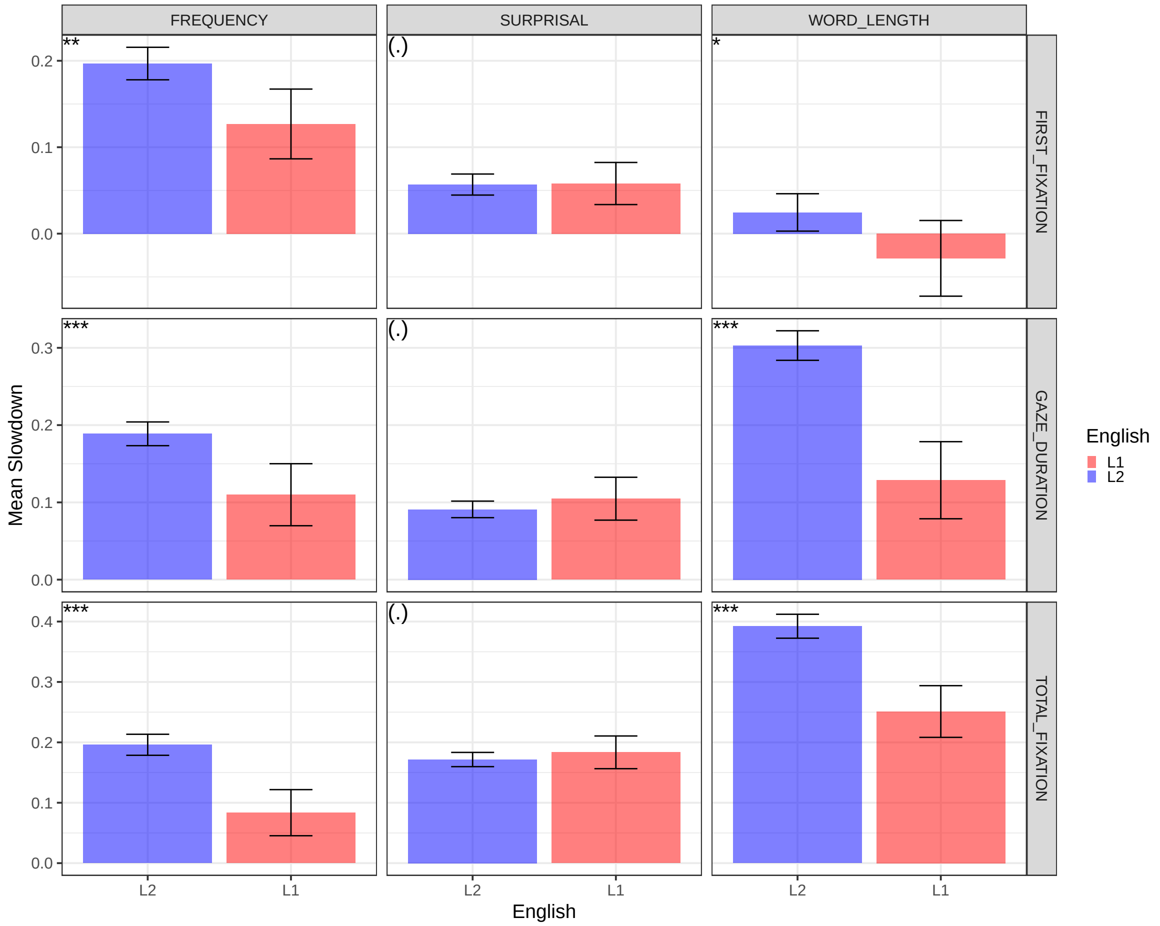

Figure S5: Mean per subject slowdown effects with 95% confidence intervals for **Z normalized reading times**, based on the GAM model in Method, fitted separately for each subject. Top left: statistical significance of a t-test for the difference between English L1 and English L2. '***' $p < 0.001$, '**' $p < 0.01$. '*' $p < 0.05$, '(.)' $p > 0.05$.

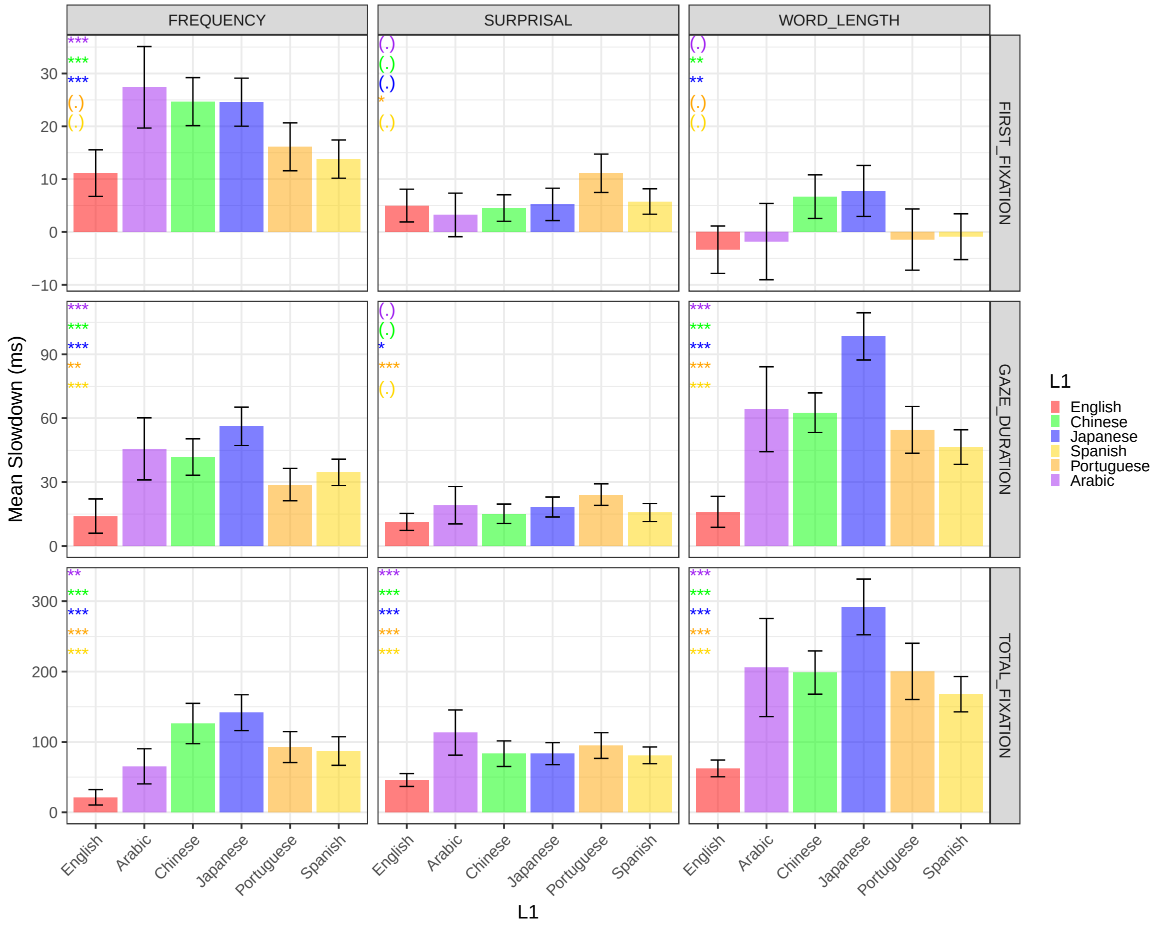

Figure S6: Mean per subject slowdown effects in *ms* by L1 with 95% confidence intervals for **raw reading times**, based on the GAM model in Method, fitted separately for each subject. Top left: statistical significance of a t-test for the difference between each of the five non-English L1s and English L1. '***' $p < 0.001$, '**' $p < 0.01$. '*' $p < 0.05$, '(.)' $p > 0.05$.

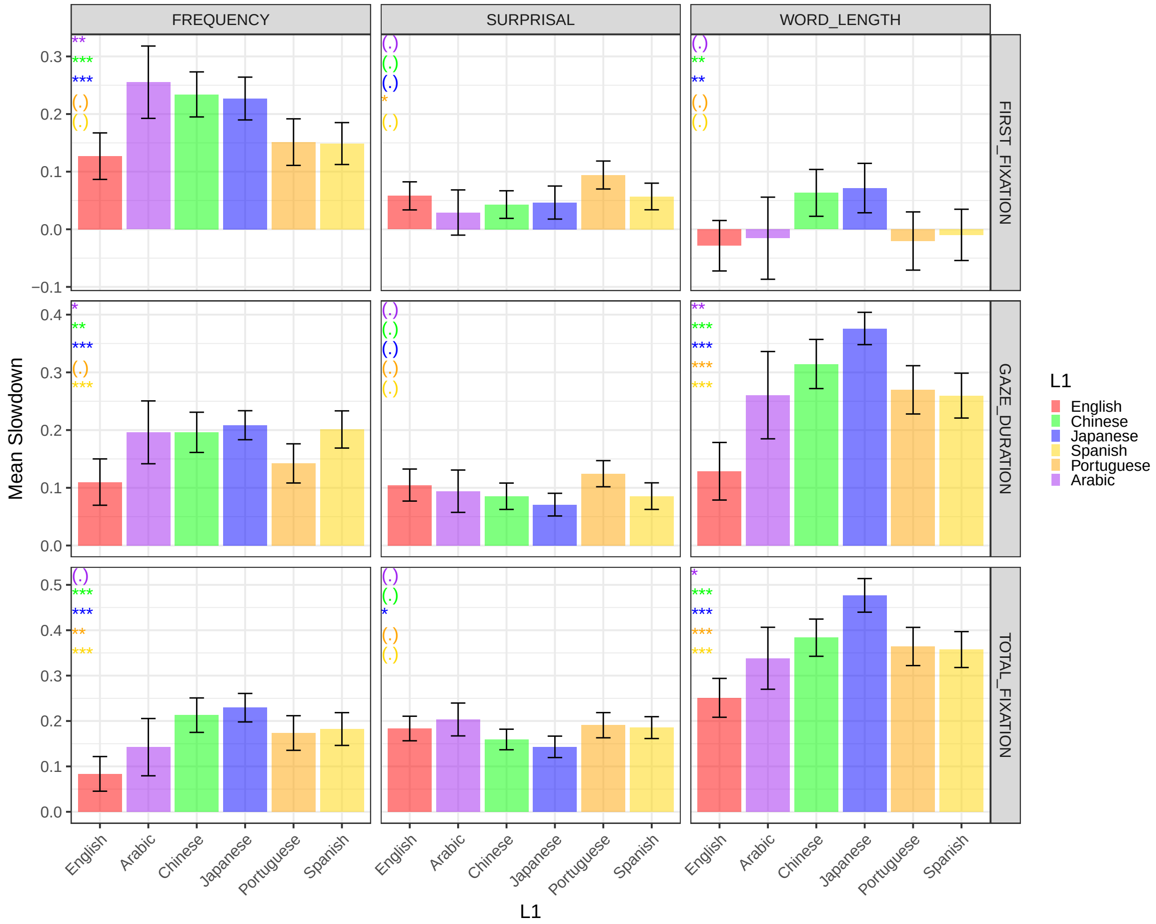

Figure S7: Mean per subject slowdown effects by L1 with 95% confidence intervals for **Z normalized reading times**, based on the GAM model in Method, fitted separately for each subject. Top left: statistical significance of a t-test for the difference between each of the five non-English L1s and English L1. '***' $p < 0.001$, '**' $p < 0.01$. '*' $p < 0.05$, '(.)' $p > 0.05$.

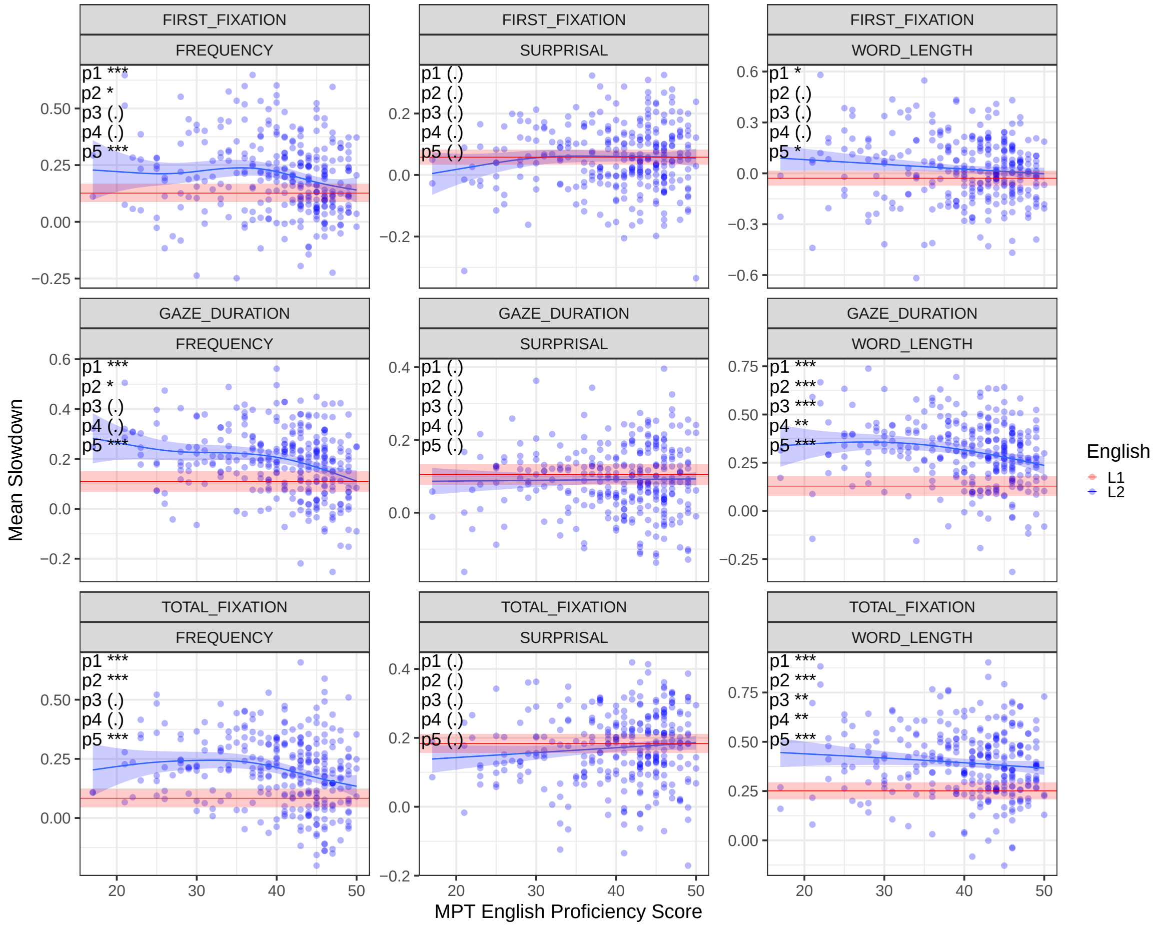

Figure S8: Slowdown effects associated with word properties of the current word for **Z normalized reading times** as a function of English proficiency. Each blue circle is a single L2 speaker. The y axis is the mean slowdown effect for the word property from a GAM model fitted separately for each participant and measure (see Methods for slowdown and GAM specifications). The x axis is the MPT English proficiency score. The blue line is a GAM fit through the L2 slowdown effects, and the red line is the mean L1 slowdown effect, both with 95% confidence intervals. Statistical significance of relevant hypothesis tests described in Method is indicated in top left. '***' $p < 0.001$, '**' $p < 0.01$. '*' $p < 0.05$, '(.)' $p > 0.05$.

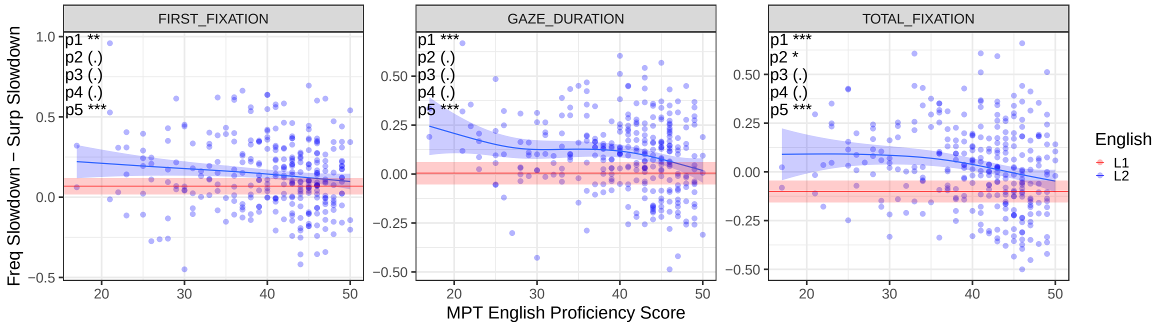

Figure S9: The frequency–surprisal slowdown effect difference for current word **Z normalized reading times**, as a function of English proficiency. Each blue circle is a single L2 speaker; curves are obtained from a GAM model specified in Method, fitted separately for each participant and fixation measure. The x axis is the MPT English proficiency score. The blue line is a GAM fit through the L2 values, and the red line is the mean of the L1 values, both with 95% confidence intervals. Statistical significance of relevant hypothesis tests described in Method is indicated in top left. '***' $p < 0.001$, '**' $p < 0.01$. '*' $p < 0.05$, '(.)' $p > 0.05$.

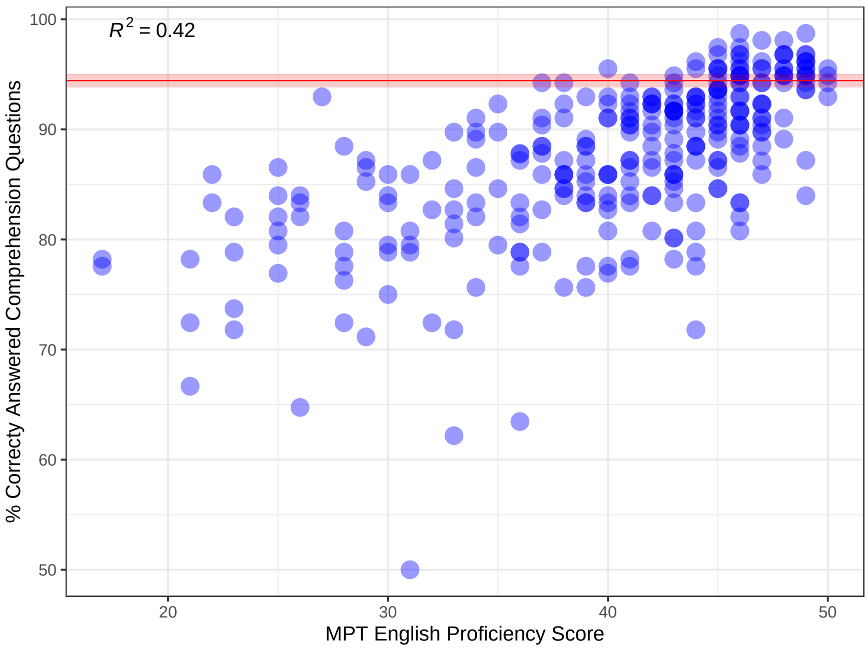

Figure S10: Percentage of correctly answered questions out of 156 questions (Shared and Individual regimes) against MPT English proficiency scores in English L2 speakers. The red horizontal line depicts the mean L1 percentage with a 95% confidence interval.

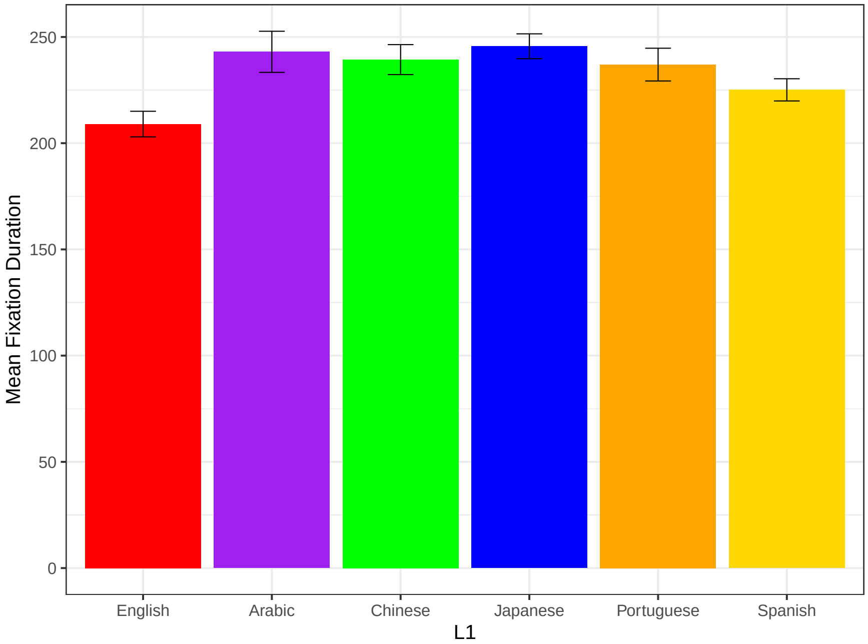

Figure S11: Mean fixation duration by L1 and 95% confidence intervals from the model $fixation\_duration \sim 1 + (1|subject)$ applied to each L1 group.

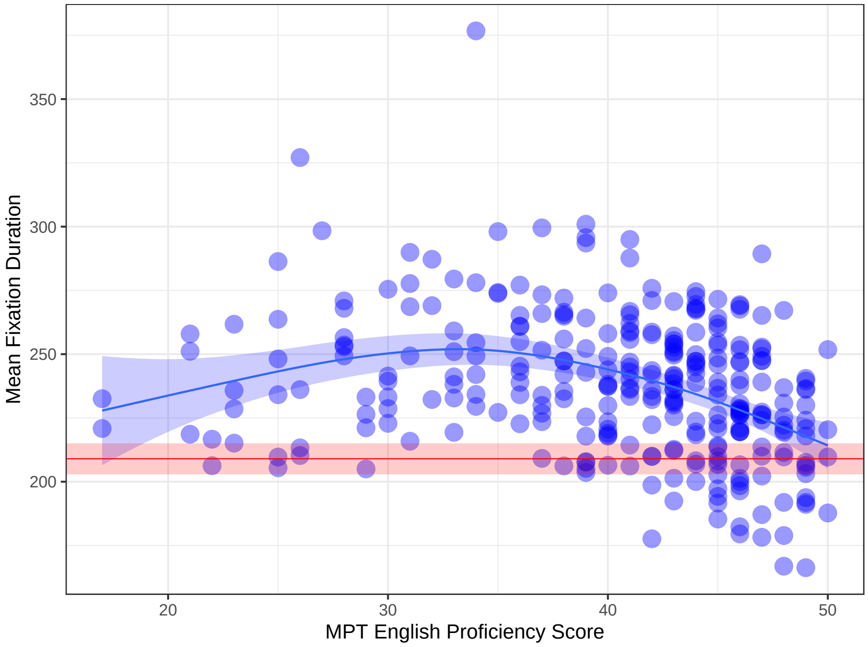

Figure S12: Mean fixation duration as a function of MPT English proficiency for English L2 participants, along with a GAM fit. Red horizontal line is the mean fixation duration for L1 speakers with 95% confidence interval.

(a) Part 1 data, 182 participants (Exploratory)

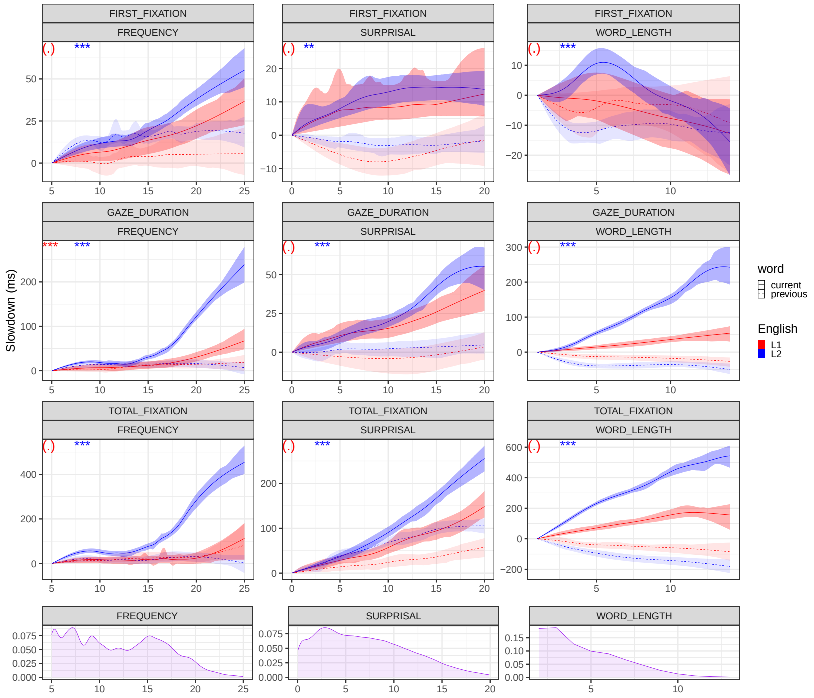

(b) Part 2 data, 183 participants (Confirmatory)

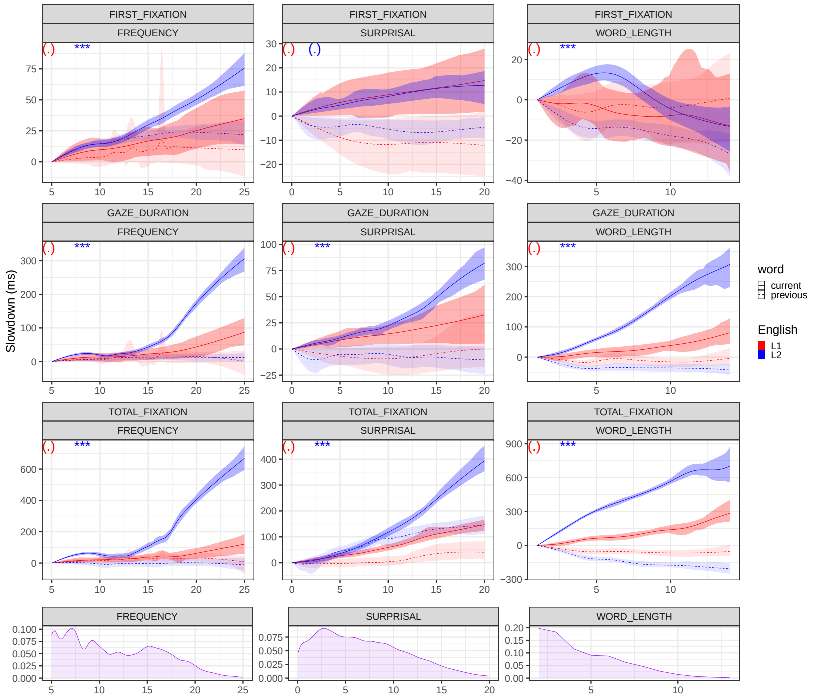

Figure S13: GAM fits for the relation between word properties and **raw reading times** in L1 and L2 speakers using the model specified in Method. Upper three rows depict slowdown effects in *ms* as a function of frequency, surprisal and word length for First Fixation, Gaze Duration and Total Fixation, with bootstrapped 95% confidence intervals. Continuous and dashed lines correspond to the word property of the current and previous word respectively. Curves are depicted in blue for L1 and in red for L2. At the top left is the significance of the quadratic term when replacing the word property smooth term of the current word with a linear and quadratic terms. '***' $p < 0.001$, '**' $p < 0.01$. '*' $p < 0.05$, '(.)' $p > 0.05$. Bottom row: Density plots for frequency, surprisal and word length values.

(a) Part 1 data, 182 participants (Exploratory)

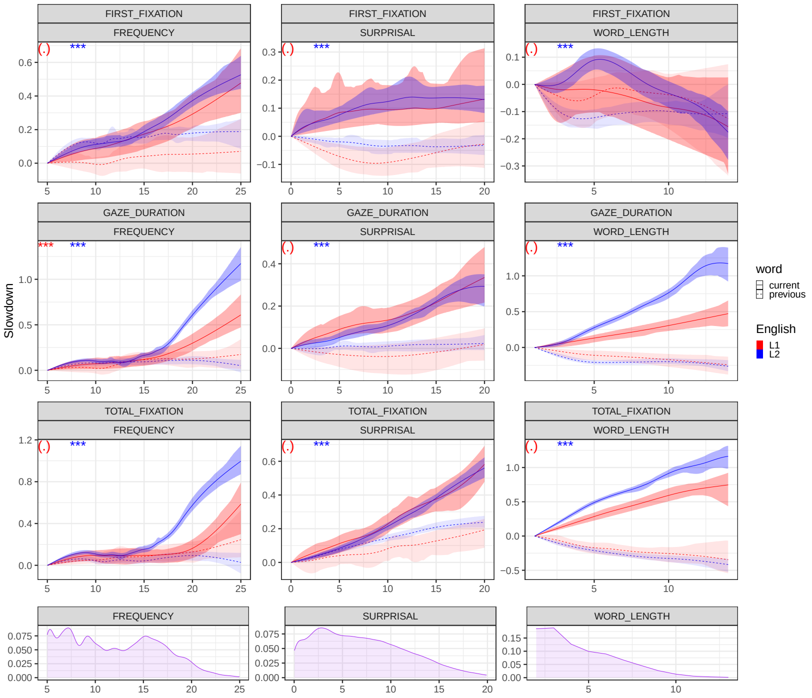

(b) Part 2 data, 183 participants (Confirmatory)

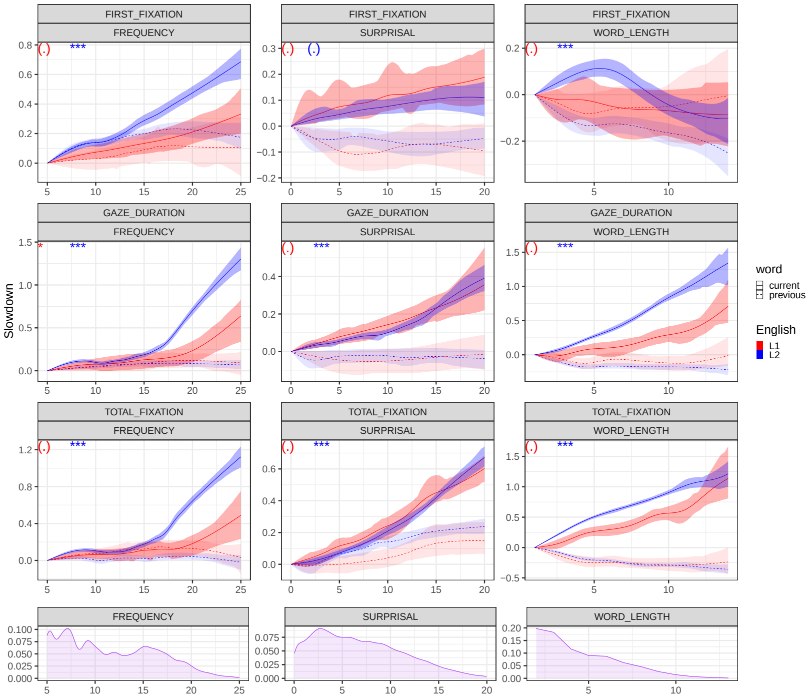

Figure S14: GAM fits for the relation between word properties and **Z normalized reading times** in L1 and L2 speakers using the model specified in Method. Upper three rows depict slowdown effects as a function of frequency, surprisal and word length for First Fixation, Gaze Duration and Total Fixation, with bootstrapped 95% confidence intervals. Continuous and dashed lines correspond to the word property of the current and previous word respectively. Curves are depicted in blue for L1 and in red for L2. At the top left is the significance of the quadratic term when replacing the word property smooth term of the current word with a linear and quadratic terms. '***' $p < 0.001$, '**' $p < 0.01$. '*' $p < 0.05$, '(.)' $p > 0.05$. Bottom row: Density plots for frequency, surprisal and word length values.

(a) Part 1 data, 182 participants (Exploratory)

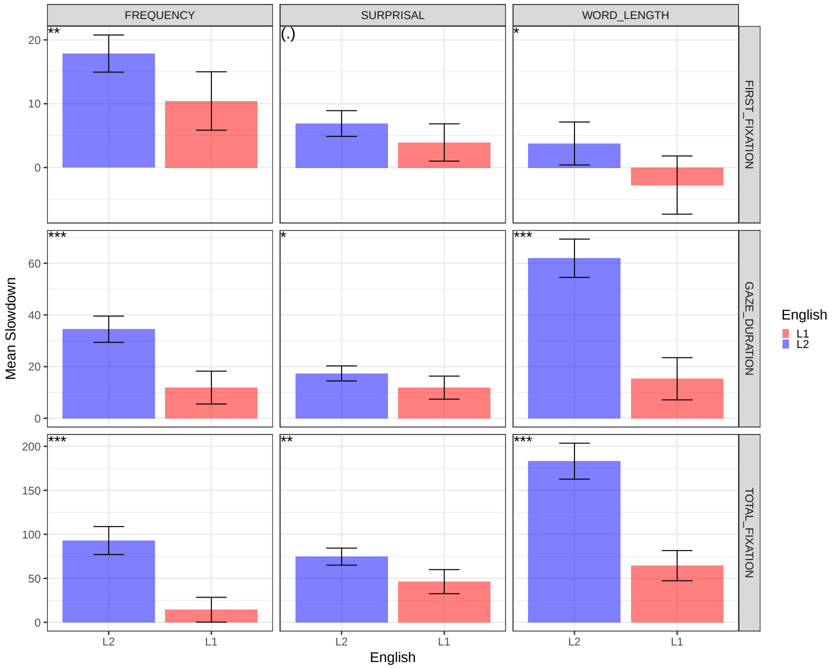

(b) Part 2 data, 183 participants (Confirmatory)

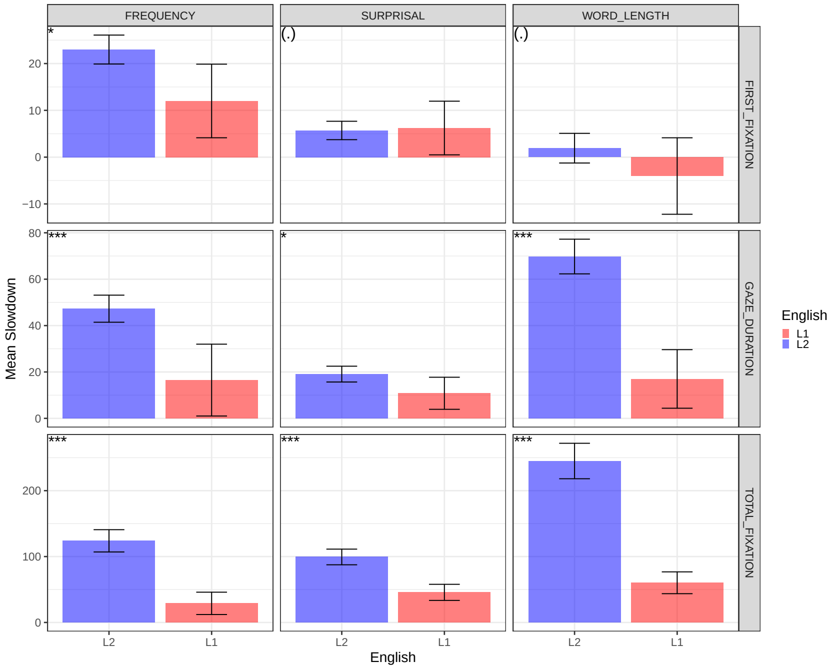

Figure S15: Mean per subject effects with 95% confidence intervals for **raw reading times**, based on the GAM model in Method, fitted separately for each subject. Top left: statistical significance of a t-test for the difference between English L1 and English L2. '***' $p < 0.001$, '**' $p < 0.01$. '*' $p < 0.05$, '(.)' $p > 0.05$.

(a) Part 1 data, 182 participants (Exploratory)

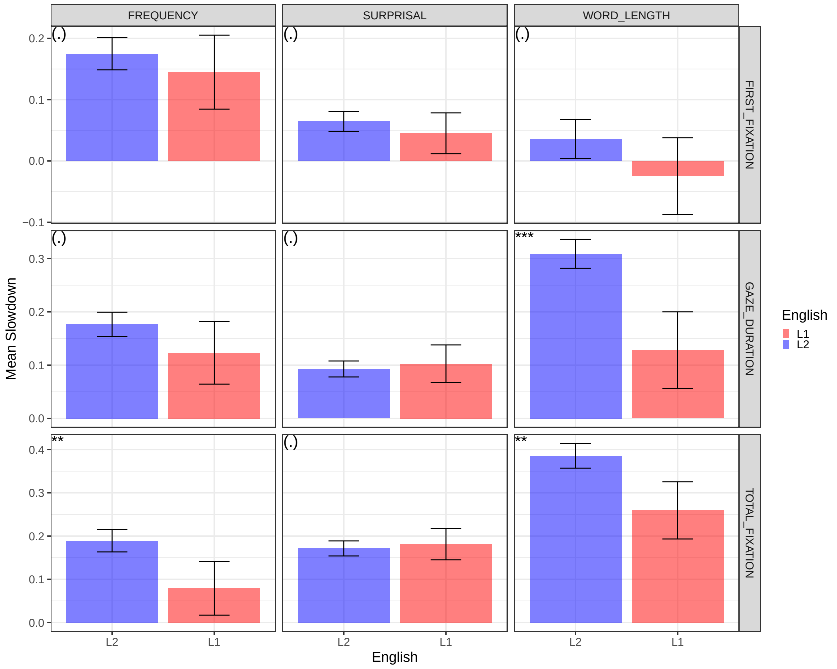

(b) Part 2 data, 183 participants (Confirmatory)

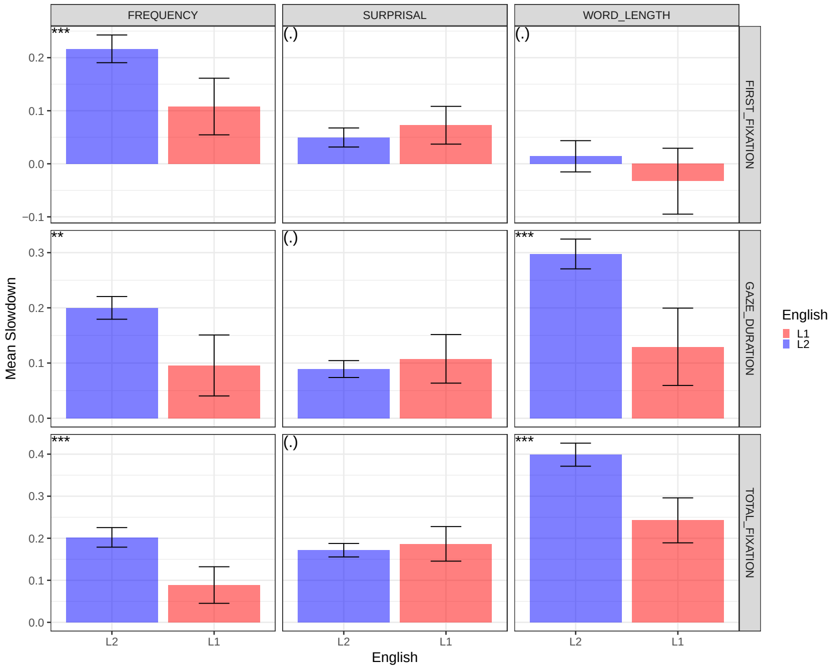

Figure S16: Mean per subject effects with 95% confidence intervals for **Z normalized reading times**, based on the GAM model in Method, fitted separately for each subject. Top left: statistical significance of a t-test for the difference between English L1 and English L2. '***' $p < 0.001$, '**' $p < 0.01$. '*' $p < 0.05$, '(.)' $p > 0.05$.

(a) Part 1 data, 182 participants (Exploratory)

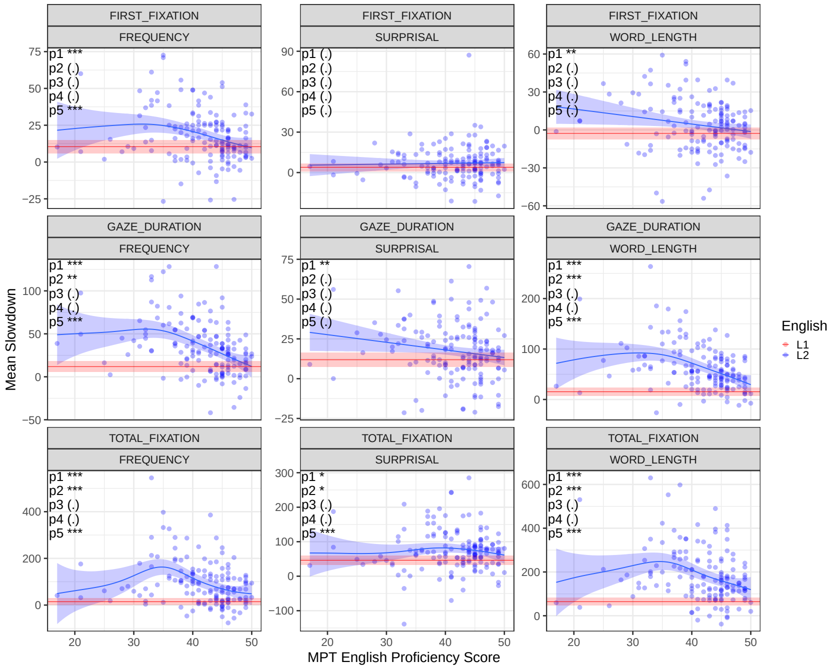

(b) Part 2 data, 183 participants (Confirmatory)

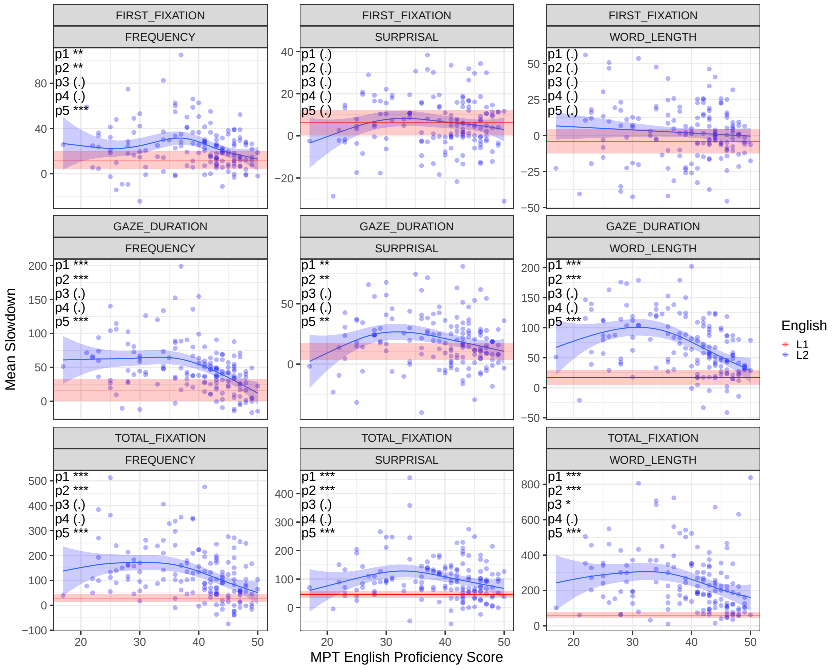

Figure S17: Slowdown effects associated with word properties of the current word for **raw reading times** as a function of English proficiency. Each blue circle is a single L2 speaker. The y axis is the mean slowdown effect for the word property from a GAM model fitted separately for each participant and measure (see Methods for slowdown and GAM specifications). The x axis is the MPT English proficiency score. The blue line is a GAM fit through the L2 slowdown effects, and the red line is the mean L1 slowdown effect, both with 95% confidence intervals. Statistical significance of relevant hypothesis tests described in Method is indicated in top left. '***' $p < 0.001$, '**' $p < 0.01$. '*' $p < 0.05$, '(.)' $p > 0.05$.

(a) Part 1 data, 182 participants (Exploratory)

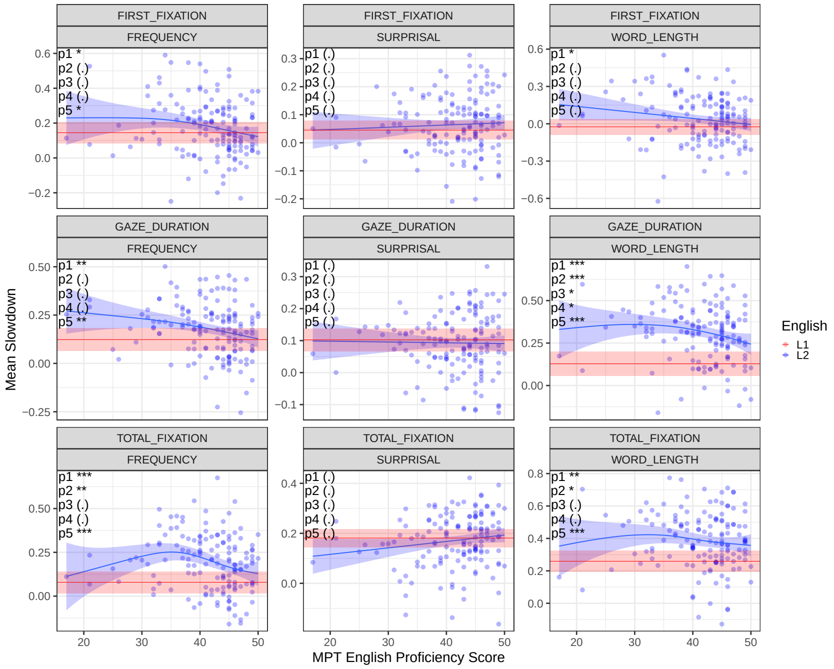

(b) Part 2 data, 183 participants (Confirmatory)

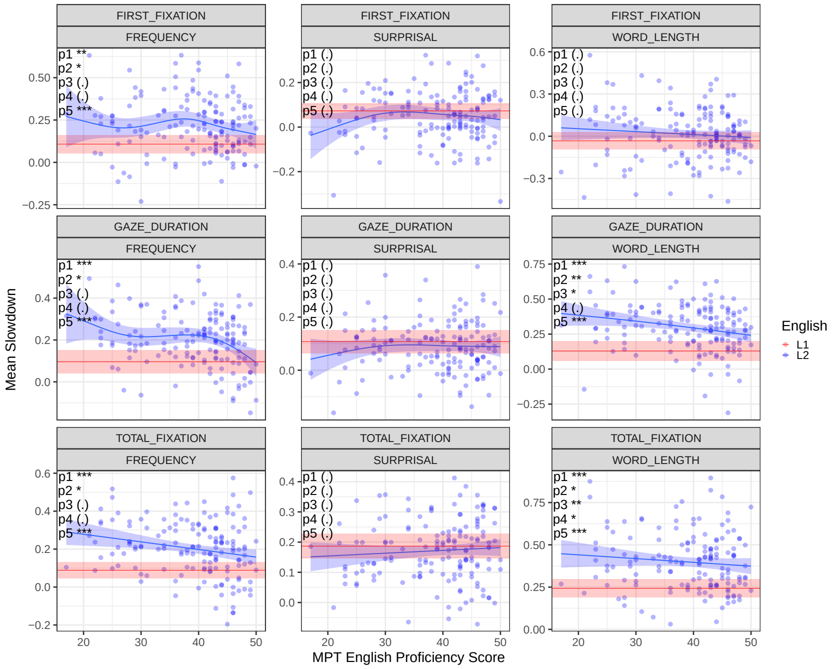

Figure S18: Slowdown effects associated with word properties of the current word for **Z normalized reading times** as a function of English proficiency. Each blue circle is a single L2 speaker. The y axis is the mean slowdown effect for the word property from a GAM model fitted separately for each participant and measure (see Methods for slowdown and GAM specifications). The x axis is the MPT English proficiency score. The blue line is a GAM fit through the L2 slowdown effects, and the red line is the mean L1 slowdown effect, both with 95% confidence intervals. Statistical significance of relevant hypothesis tests described in Method is indicated in top left. '***' $p < 0.001$, '**' $p < 0.01$. '*' $p < 0.05$, '(.)' $p > 0.05$.

(a) Part 1 data, 182 participants (Exploratory)

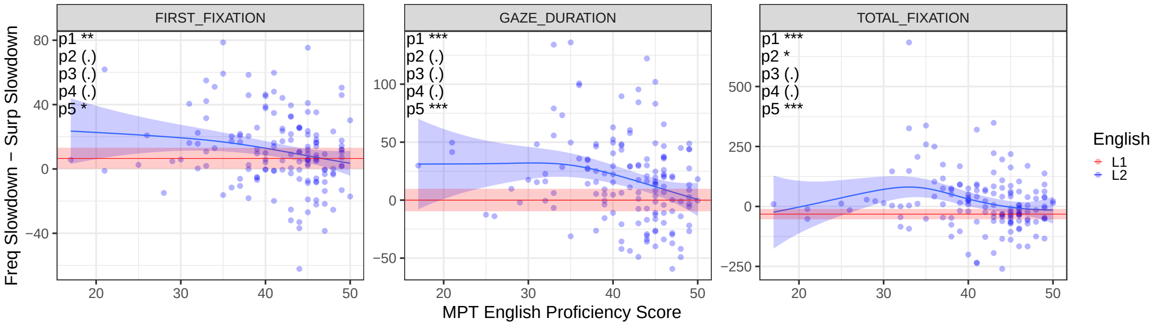

(b) Part 2 data, 183 participants (Confirmatory)

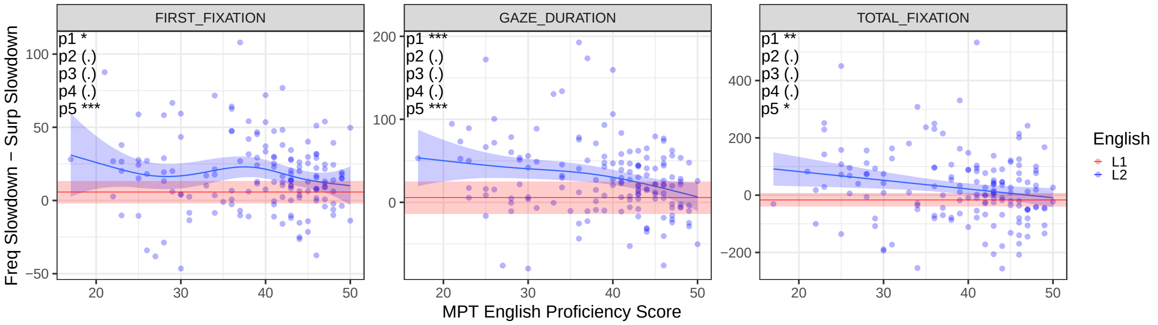

Figure S19: The frequency–surprisal slowdown effect difference for current word **raw reading times**, as a function of English proficiency. Each blue circle is a single L2 speaker; curves are obtained from a GAM model specified in Method, fitted separately for each participant and fixation measure. The x axis is the MPT English proficiency score. The blue line is a GAM fit through the L2 values, and the red line is the mean of the L1 values, both with 95% confidence intervals. Statistical significance of relevant hypothesis tests described in Method is indicated in top left. '***' $p < 0.001$, '**' $p < 0.01$. '*' $p < 0.05$, '(.)' $p > 0.05$.

(a) Part 1 data, 182 participants (Exploratory)

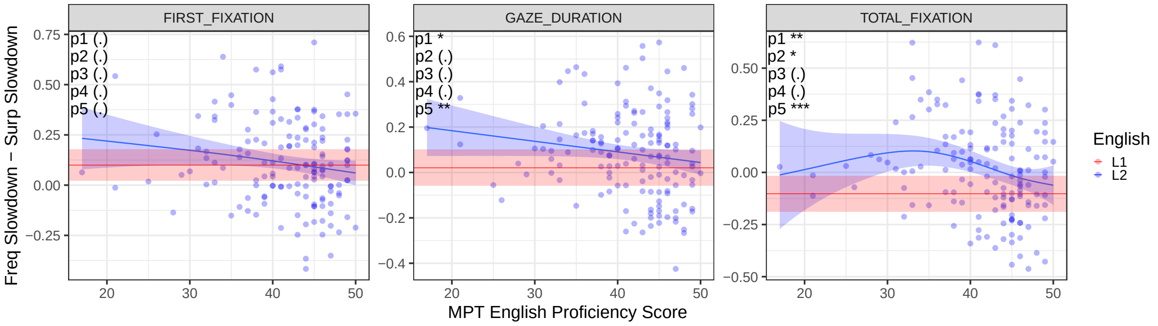

(b) Part 2 data, 183 participants (Confirmatory)

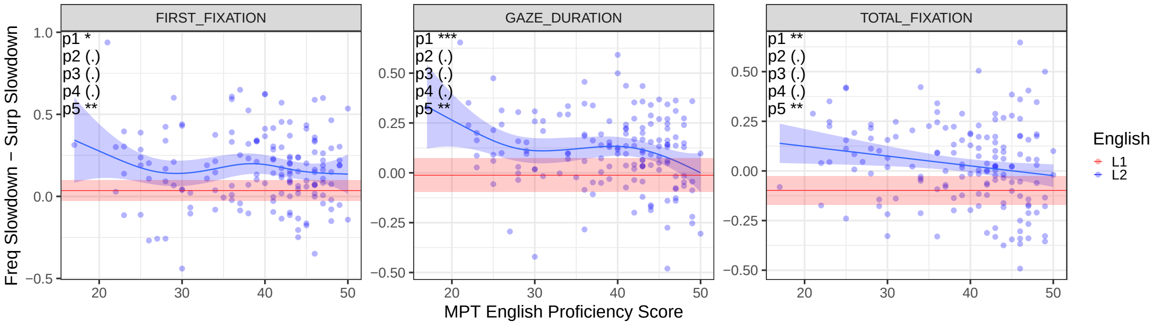

Figure S20: The frequency–surprisal slowdown effect difference for current word **Z normalized reading times**, as a function of English proficiency. Each blue circle is a single L2 speaker; curves are obtained from a GAM model specified in Method, fitted separately for each participant and fixation measure. The x axis is the MPT English proficiency score. The blue line is a GAM fit through the L2 values, and the red line is the mean of the L1 values, both with 95% confidence intervals. Statistical significance of relevant hypothesis tests described in Method is indicated in top left. '***' $p < 0.001$, '**' $p < 0.01$. '*' $p < 0.05$, '(.)' $p > 0.05$.

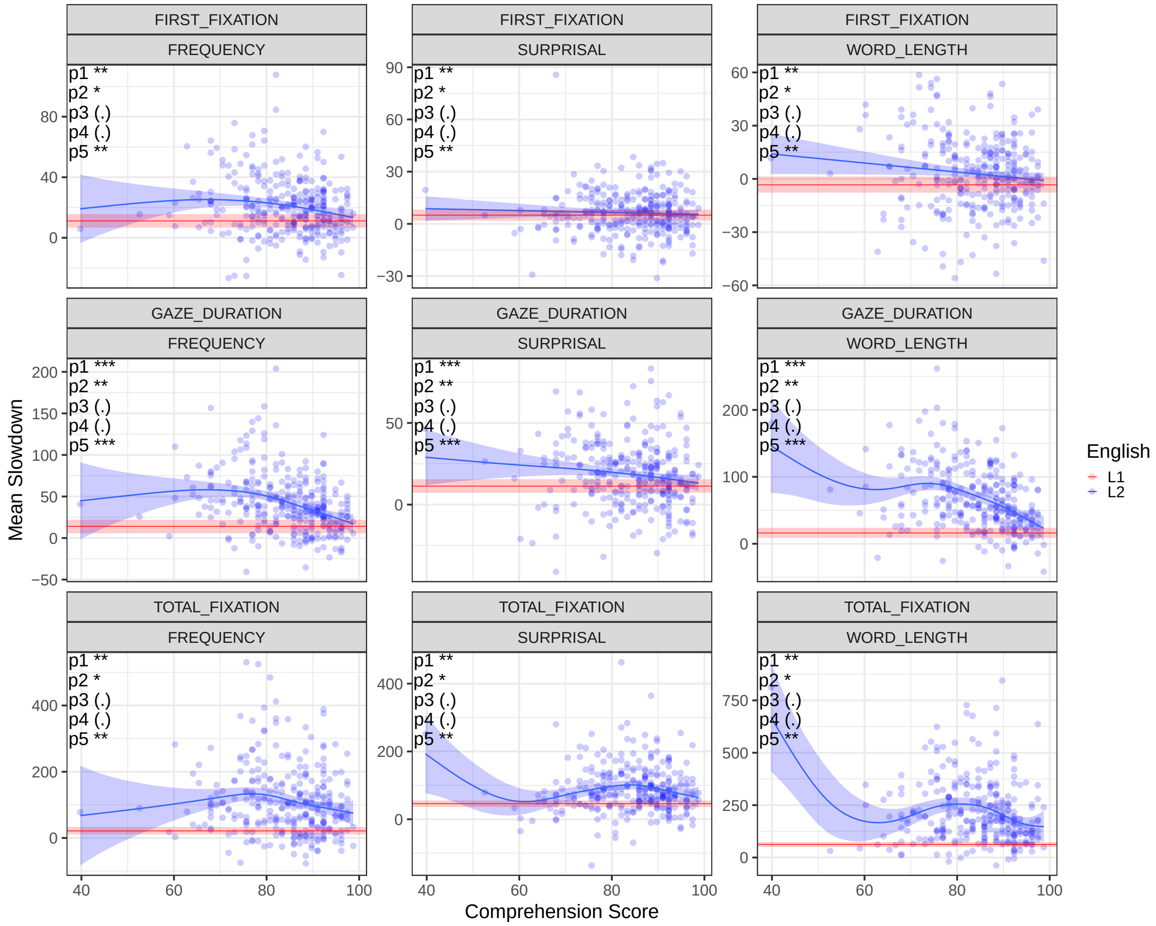

Figure S21: Slowdown effects associated with word properties of the current word for **raw reading times** as a function of reading comprehension. Each blue circle is a single L2 speaker. The y axis is the mean slowdown effect for the word property from a GAM model fitted separately for each participant and measure (see Methods for slowdown and GAM specifications). The x axis is the percentage of correctly answered reading comprehension questions in the Shared Regime, out of a total of 78 questions. The blue line is a GAM fit through the L2 slowdown effects, and the red line is the mean L1 slowdown effect, both with 95% confidence intervals. Statistical significance of relevant hypothesis tests described in Method is indicated in top left. '***' $p < 0.001$, '**' $p < 0.01$. '*' $p < 0.05$, '(.)' $p > 0.05$.

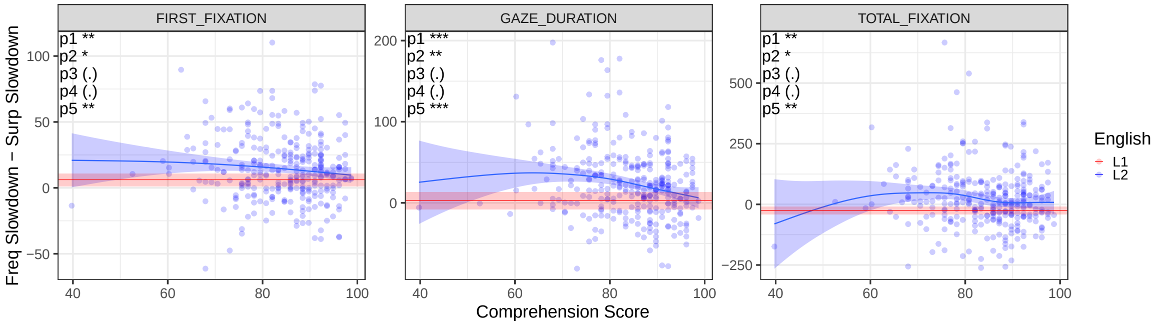

Figure S22: The frequency–surprisal slowdown effect difference for current word **raw reading times**, as a function of reading comprehension. Each blue circle is a single L2 speaker; curves are obtained from a GAM model specified in Method, fitted separately for each participant and fixation measure. The x axis is the percentage of correctly answered reading comprehension questions, out of a total of 78 questions in the Shared Regime. The blue line is a GAM fit through the L2 values, and the red line is the mean of the L1 values, both with 95% confidence intervals. Statistical significance of relevant hypothesis tests described in Method is indicated in top left. '***' $p < 0.001$, '**' $p < 0.01$. '*' $p < 0.05$, '(.)' $p > 0.05$.
